# Impact of HCV Testing and Treatment on HCV Transmission Among Men Who Have Sex With Men and Who Inject Drugs in San Francisco: A Modelling Analysis

**DOI:** 10.1093/infdis/jiad169

**Published:** 2023-07-24

**Authors:** Adelina Artenie, Jack Stone, Shelley N Facente, Hannah Fraser, Jennifer Hecht, Perry Rhodes, Willi McFarland, Erin Wilson, Matthew Hickman, Peter Vickerman, Meghan D Morris

**Affiliations:** Population Health Sciences, Bristol Medical School, University of Bristol, Bristol, United Kingdom; Population Health Sciences, Bristol Medical School, University of Bristol, Bristol, United Kingdom; School of Public Health, University of California Berkeley, Berkeley, California, USA; Facente Consulting, Richmond, California, USA; Population Health Sciences, Bristol Medical School, University of Bristol, Bristol, United Kingdom; San Francisco AIDS Foundation, San Francisco, California, USA; Springboard HealthLab, Berkeley, California, USA; Facente Consulting, Richmond, California, USA; University of California San Francisco Alliance Health Project, San Francisco, California, USA; San Francisco Department of Public Health, San Francisco, California, USA; San Francisco Department of Public Health, San Francisco, California, USA; Population Health Sciences, Bristol Medical School, University of Bristol, Bristol, United Kingdom; Population Health Sciences, Bristol Medical School, University of Bristol, Bristol, United Kingdom; Department of Epidemiology and Biostatistics, University of California San Francisco, San Francisco, California, USA

**Keywords:** HCV elimination, HIV, MSM, MSM-IDU, hepatitis C

## Abstract

**Background:**

Men who have sex with men who ever injected drugs (ever MSM-IDU) carry a high hepatitis C virus (HCV) burden. We estimated whether current HCV testing and treatment in San Francisco can achieve the 2030 World Health Organization (WHO) HCV elimination target on HCV incidence among ever MSM-IDU.

**Methods:**

A dynamic HCV/HIV transmission model among MSM was calibrated to San Francisco data, including HCV antibody (15.5%, 2011) and HIV prevalence (32.8%, 2017) among ever MSM-IDU. MSM had high HCV testing (79%–86% ever tested, 2011–2019) and diagnosed MSM had high HCV treatment (65% ever treated, 2018). Following coronavirus disease 2019 (COVID-19)–related lockdowns, HCV testing and treatment decreased by 59%.

**Results:**

Among all MSM, 43% of incident HCV infections in 2022 were IDU-related. Among ever MSM-IDU in 2015, HCV incidence was 1.2/100 person-years (95% credibility interval [CrI], 0.8–1.6). Assuming COVID-19–related declines in HCV testing/treatment persist until 2030, HCV incidence among ever MSM-IDU will decrease by 84.9% (95% CrI, 72.3%–90.8%) over 2015–2030. This decline is largely attributed to HCV testing and treatment (75.8%; 95% CrI, 66.7%–89.5%). Slightly greater decreases in HCV incidence (94%–95%) are projected if COVID-19 disruptions recover by 2025 or 2022.

**Conclusions:**

We estimate that HCV incidence will decline by >80% over 2015–2030 among ever MSM-IDU in San Francisco, achieving the WHO target.

## Teaser

Modelling projects that HCV incidence will decrease by ≥80% over 2015–2030 (thus, achieving the WHO elimination target) among men who have sex with men and who inject drugs in San Francisco, largely due to high HCV testing and treatment.

**(See the Editorial Commentary by Fierer and Schinkel on pages 657–61.)**

The development of highly curative direct-acting antiviral (DAA) therapy for hepatitis C virus (HCV) infection prompted the World Health Organization (WHO) to call for global elimination of HCV as a public health problem by 2030 [1], including the key target of an 80% reduction in HCV incidence over 2015–2030 [1].

To achieve the WHO elimination goal, it is essential to scale-up HCV testing and treatment [1]. With 3 times the prevalence of the general population [2], men who have sex with men (MSM) represent a key target group for eliminating HCV [1]. Studies estimate that 10%–20% of MSM have ever injected drugs (MSM-IDU) [3–5], with 6%–14% in the last year [6, 7]. In a recent global systematic review, MSM-IDU had substantially higher HCV prevalence (30.2%) than MSM who never injected drugs (MSM non-IDU; 2.7%) [2]. Several studies among MSM have found injection drug use (IDU) to carry one of the highest risks of HCV infection, although sexual practices (eg, unprotected receptive anal intercourse) also heighten risk [8–10]. In San Francisco, survey data from 2011 suggest a much higher HCV antibody prevalence among MSM-IDU (15.5%) than MSM non-IDU (2.3%) [5]. Together, these findings highlight the importance of IDU for HCV transmission among MSM. However, previous epidemic modelling studies among MSM [11–16] have focused only on sexual transmission of HCV infection or included a generic high-risk group, thus omitting to evaluate whether HCV services adequately serve MSM-IDU.

Building upon prior multisector collaborative efforts to reduce HIV, San Francisco introduced the first city-focused strategic plan to eliminate HCV in the United States. Started in 2016, End Hep C SF is an initiative of the public health department, research university, and community partners, which collaborates around prevention, testing, and care activities, prioritizing communities hardest hit by HCV, including MSM and people who use drugs [17]. Locally, surveillance of human immunodeficiency virus (HIV), HCV, risk behaviors and services access among MSM has been ongoing since 2004 through the National HIV Behavioral Surveillance (NHBS), and more recently, also using the street-intercept surveys led by the San Francisco AIDS Foundation (SFAF-S) [5, 18–20]. In recent years, there have been several efforts to characterize the epidemiology of HCV in MSM and other risk groups to inform planning for HCV elimination [21, 22]. However, despite this progress, San Francisco experienced decreases in HCV testing and treatment during the coronavirus disease 2019 (COVID-19) pandemic [23, 24]. We used epidemic modelling to evaluate decreases in HCV incidence achieved among MSM-IDU and to assess whether elimination would be reached by 2030 for different scenarios of how HCV testing and treatment rates recovered after the pandemic.

## METHODS

### Model Description

**Figure 1.**
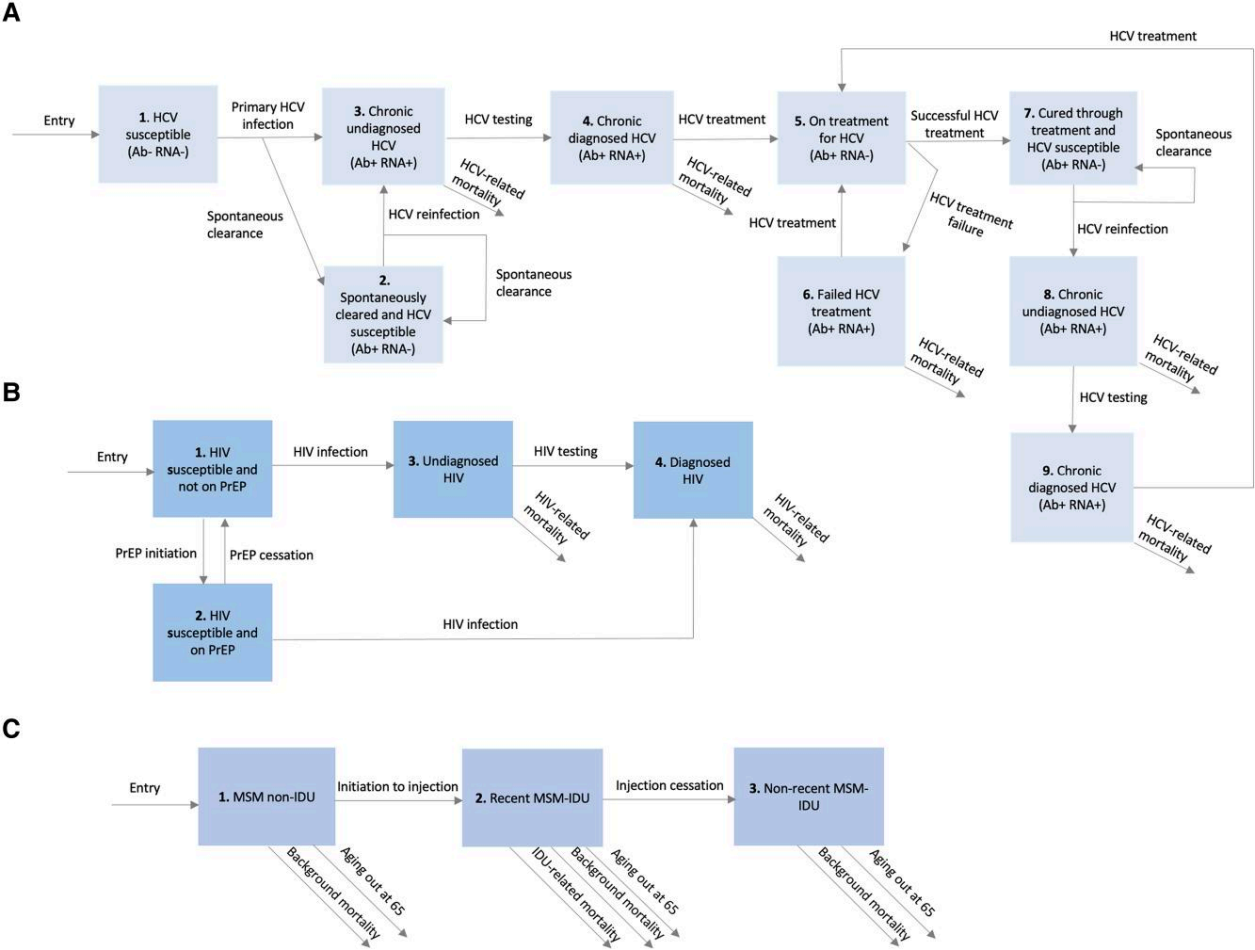
Model schematic illustrating the HCV infection compartments (*A*), HIV infection compartments (*B*), and injection drug use compartments (*C*). Background mortality includes non-HIV, non-HCV, and non-IDU-related death. Abbreviations: Ab, antibody; HCV, hepatitis C virus; HIV, human immunodeficiency virus; IDU, injection drug use; MSM, men who have sex with men; PrEP, preexposure prophylaxis.

We developed a dynamic deterministic HCV and HIV transmission model among MSM in San Francisco, including transmission through IDU and sexual risk behaviors. MSM are stratified by HCV and HIV infection status and history of IDU (Figure 1). Specifically, MSM are stratified by whether they have injected in the last year (recent MSM-IDU) or previously (nonrecent MSM-IDU), or never injected drugs (MSM non-IDU). We refer to MSM who injected recently or nonrecently as ever MSM-IDU. The majority of MSM-IDU in San Francisco primarily inject meth/amphetamine [7].

Individuals enter the model at age 15 years susceptible to HCV and HIV with no IDU history and not on HIV preexposure prophylaxis (PrEP). Entry into the model is set to balance (1) injection-related mortality (recent MSM-IDU only), (2) other non-HIV/HCV related death, and (3) aging out at age 65 years. Ages 15 and 65 years were set to reflect the onset and end of sexual activity [16]. MSM also exit the model due to HIV- or HCV-related mortality. Individuals initiate and cease injecting at fixed rates, with only permanent cessation of injecting being modelled.

HIV and HCV transmission through IDU only occurs between recent MSM-IDU contacts, while sexual HIV and HCV transmission can occur between any MSM contacts. Mixing to form these contacts is assumed to occur randomly. Based on data indicating similar numbers of sexual partners and condom use by injecting status (Supplementary Material, page 3), sexual HIV and HCV transmission risk was assumed to not differ by injecting status.

The risk of sexual HCV transmission depends on the chronic HCV prevalence among all MSM, while injecting HCV transmission risk depends on the chronic HCV prevalence among MSM-IDU. HCV infectivity is elevated among MSM who are HIV-HCV coinfected. Once infected with HCV, a proportion of MSM spontaneously clear infection, while the remainder develop undiagnosed chronic infection. Chronically infected MSM can undergo HCV testing, and once diagnosed, can initiate treatment, which can result in a sustained virological response (SVR). Treatment efficacy and duration depend on the type of treatment (classified as pegylated interferon or DAAs). Those who do not achieve SVR remain chronically infected. Cured individuals can become reinfected, diagnosed, and treated again at the same rates related to their risk groups, regardless of prior infection and treatment history (no US restrictions exist on retreatment). In the overall MSM population, HCV reinfection is higher than primary HCV due to heterogeneity in HCV risk by IDU status.

The risk of injecting and sexual HIV transmission depends on the HIV prevalence among MSM-IDU and all MSM, respectively. From 2010, MSM can increasingly initiate PrEP and can cease PrEP at a fixed rate. Once infected with HIV, MSM not on PrEP are assumed to have undiagnosed infection. These MSM can undergo HIV testing, becoming diagnosed, with a time-varying proportion of diagnosed MSM receiving antiretroviral therapy (ART). MSM on ART have reduced HIV infectivity. Susceptible MSM on PrEP have a lower risk of HIV acquisition [25, 26] compared to those not on PrEP, and if infected, move directly to the diagnosed group due to frequent HIV testing [27].

### Model Parameterization and Calibration

The model was parameterized and calibrated to San Francisco data, based on published literature and data analyses done on NHBS-MSM, SFAF-S, and medical record data at the community health center Strut (Supplementary Table 1). Key parameters are summarized in Table 1 and details provided in the Supplementary Material (pages 3–7 and Supplementary Table 3). In Table 2, we summarize HCV and HIV testing and treatment data used to parametrize and calibrate the model. For all parameters, we assumed they remained constant after the last available data, except for rates of HCV and HIV testing and treatment and PrEP initiation, which decreased due to COVID-19–related disruptions. In brief, HCV testing started between 1999 and 2001 [5, 19] and increased linearly until 2017 to calibrate to data on the proportion of MSM reporting past-year HCV testing by IDU and HIV statuses, and to reflect high levels of HCV testing (approximately 80%) as early as 2004 [5, 19] (NHBS-MSM and SFAF-S, unpublished). Compared to MSM without HIV non-IDU not on PrEP, rates of HCV testing for all other MSM were parameterized to be 2.5-fold (95% confidence interval [CI], 1.8–3.5) greater (NHBS-MSM, unpublished); aligning with other data [38] and US testing guidelines [39]. Interferon-based HCV treatment was started in 2002–2004 [32] and scaled-up linearly until 2012 to calibrate to data on the proportion of MSM with HIV receiving treatment [34], then remaining stable over 2012–2014. In 2015, following the introduction of DAAs [40], we assumed the HCV treatment rate was scaled-up, remaining stable afterwards to calibrate to the proportion of MSM reporting ever treatment in 2018 (63.6%; 95% CI, 45.1%–79.6%; NHBS-MSM and SFAF-S, unpublished). The same HCV treatment rates were assumed among all diagnosed MSM [41, 42].

**Table 1.** Prior and Posterior Ranges for Key Parameters Used in the Model^a^

Parameter	Priors: Distribution and Estimates	Sources and Comments	Posterior Ranges, Median (Min/Max)
MSM population size in 1985	Uniform: 65 523–140 000	The lower bound is the lower bound of the 2017 estimate of the MSM population size in San Francisco [21] We set the upper bound higher than the 2017 estimate, as the population is expected to have decreased since 1985 due to HIV-related mortality [28]	132 560 (123 250–139 980)
Sexually related HCV transmission rate, per person per year	Uniform: 0–0.5	Uninformative prior; varied to calibrate to HCV prevalence	0.03 (0.02–0.04)
RR of HCV transmission through injection drug use practices vs sexual practices	Uniform: 2–10	Wide range informed by studies estimating the magnitude of the associations between IDU and sexual risk behaviors and incident or prevalent HCV infection (Supplementary Table 2); varied to calibrate to HCV prevalence	2.8 (2.0–3.8)
RR of HCV infectivity among MSM with HIV vs MSM without HIV	Lognormal: 2.6 (95% CI, 1.5–4.4)	In the absence of MSM-specific data, it is based on a systematic review and meta-analysis comparing the risk of mother-to-child HCV transmission in mothers with and without HIV [29] This finding is corroborated by 2 other studies examining the probability of needlestick HCV transmission (Supplementary Table 3)	2.1 (1.5–2.8)
Proportion of individuals who spontaneously clear HCV infection among MSM without HIV	Uniform: 0.22–0.29	[30]	0.24 (0.22–0.26)
Proportion of individuals who spontaneously clear HCV infection among MSM with HIV	Uniform: 0.12–0.19	[31]	0.17 (0.12–0.19)
Year HCV testing started	Uniform: 1999–2001	Assumption based on the high level of HCV testing reported by MSM enrolled in NHBS-MSM 2004 and 2008 [19], which reflects the earliest years for which data are available Details are provided in the Supplementary Material (section 1.2.3, page 4)	1999.2 (1999.0–1999.5)
Proportion of MSM non-IDU, without HIV, and not on PrEP that reported HCV testing in the past year	Normal: 17.3% (12.2%–23.4%)	Based on NHBS-MSM 2017 (unpublished data) Used to inform the rate of HCV testing Details are provided in the Supplementary Material (section 1.2)	17.3% (13.3% – 19.9%)
RR of HCV testing among MSM who belonged to any of the following groups, compared to MSM non-IDU, without HIV, and not on PrEP •ever MSM-IDU or •MSM without HIV on PrEP or •MSM with HIV	Lognormal: 2.5 (1.8–3.5)	Based on differences in HCV testing by injection and HIV infection statues among MSM in NHBS-MSM 2017 (unpublished) Details are provided in the Supplementary Material (section 1.2, page 3)	2.4 (1.9–2.9)
Year interferon-based HCV treatment started	Uniform: 2002–2004	[32]	2002.6 (2002.0–2003.3)
Year DAAs were introduced	2015	[33]	…
Proportion of MSM with HIV who reported ever HCV treatment over 2008–2014	Lognormal 15.7% (12.8%–18.9%)	[34] Used to inform the rate of HCV treatment before the introduction of DAAs	15.4% (13.4%–17.9%)
RR for the increase in rate of HCV treatment in 2015 due to DAA scale-up relative to previous years	Uniform 1–10	Uninformative prior	7.0 (4.0–9.8)
Rate of exit from the HCV treatment compartment during the interferon era, 2004–2014; per person per year	Point estimate: 52/48	Taken as the inverse of the average duration of treatment with interferon: 48 wk	…
Rate of exit from the HCV treatment compartment during the DAA era, 2015 onward; per person per year	Uniform: 52/8 to 52/12	Taken as the inverse of the average duration of treatment with DAAs: 8–12 wk	5.1 (4.3–6.2)
Proportion of HCV treatments that result in SVR during the interferon era, 2004–2014, among MSM without HIV	Normal: 64% (59%– 69%)	Observational study examining the efficacy of interferon- and ribavirin-based HCV treatment in participants without HIV [35]	63.7% (60.7%–67.0%)
Proportion of HCV treatments that result in SVR during the PEG-IFN era, 2004–2014, among MSM with HIV	Normal: 38% (35%–42%)	Meta-analysis of observational studies examining the efficacy of interferon- and ribavirin-based HCV treatment in participants with HIV [36].	38.1% (37.2%–38.8%)
Proportion of HCV treatments that result in SVR during the DAA era, 2015 onward, irrespective of HIV status	Uniform: 90%–100%	Based on a review of observational studies on the efficacy of DAAs [37]	97.5% (93.8%–100.0%)

Abbreviations: CI, confidence interval; DAA, direct-acting antiviral; HCV, hepatitis C virus; HIV, human immunodeficiency virus; IDU, injection drug use; MSM, men who have sex with men; NHBS, National HIV Behavioral Surveillance; PEG-IFN, pegylated interferon; PrEP, preexposure prophylaxis; RR, relative risk ;SVR, sustained virologic response.

A full list of parameters, sources and posterior ranges are provided in Supplementary Tables 3 and 12.

**Table 2.** Summary of Data on HCV and HIV Services Uptake Among MSM in San Francisco Used to Parameterize and Calibrate the Model

HCV/HIV Service	Description	Data^a^
HCV testing	Data from NHBS-MSM 2017 show higher HCV testing in MSM-IDU than MSM non-IDU Data from NHBS-MSM and SFAF MSM street-intercept study conducted over time indicate high levels of HCV testing in MSM as early as 2004	The proportion of MSM reporting past-year HCV testing in 2017 was 32% This overall estimate varied between 17% and 60% according to IDU, HIV, PrEP statuses: (1) MSM who also reported IDU, (2) people with HIV, or (3) people without HIV and on PrEP had 2.5-times higher levels of past-year HCV testing than MSM who did not report any of these exposures The proportion of MSM ever tested for HCV over 2011–2019 ranged between 79% and 86% The proportion of MSM with HIV who tested HCV Ab positive and were aware of their HCV infection over 2004–2011 was 71%–90% [5, 19]
HCV treatment	Data from NHBS-MSM and SFAF MSM street-intercept study indicate that the proportion of MSM who were ever treated for HCV was high	The proportion of MSM ever diagnosed with chronic HCV who indicate ever being treated for HCV over 2017–2019 ranged between 57% and 75%
HIV testing	Data from NHBS-MSM conducted over time indicate high levels of HIV testing as early as 2004	The proportion of MSM with HIV who indicated being diagnosed for their HIV infection increased from 78% in 2004 to 96% in 2017[18] The proportion of MSM without HIV who reported being tested for HIV in the previous year was 82% in 2017 Levels of past-year HIV testing were similar among MSM-IDU and MSM non-IDU
PrEP initiation	Data from NHBS-MSM and SFAF MSM street-intercept study conducted over time indicate that the proportion of MSM without HIV receiving PrEP has gradually increased since 2010	The proportion of MSM without HIV reporting being on PrEP increased from 1% in 2011 to 45% in 2019 [20] Levels of PrEP use were similar among MSM-IDU and MSM non-IDU
HIV treatment	Data from NHBS-MSM and SFAF MSM street-intercept study conducted over time indicate high levels of HIV treatment as early as 2008	The proportion of MSM with HIV who reported being on ART ranged between 79% and 96% over 2008–2019 [18] Levels of HIV treatment were similar among MSM-IDU and MSM non-IDU

Abbreviations: HCV, hepatitis C virus; HIV, human immunodeficiency virus; IDU, injection drug use; MSM, men who have sex with men; NHBS, National HIV Behavioral Surveillance; PrEP, preexposure prophylaxis; SFAF, San Francisco AIDS Foundation.

Only point estimates are presented. Uncertainty ranges and additional details on model parameterization and analyses of unpublished data are presented in the Supplementary Material page 3–7 and Supplementary Table 3. In the absence of a reference, data reflect unpublished estimates.

The model was also calibrated to several other data, including HCV antibody and HIV prevalence, proportions of MSM that recently/ever injected, and PrEP use (Supplementary Table 4). Calibration was performed using an approximate Bayesian computation sequential Monte Carlo scheme, accounting for uncertainty in the calibration data and parameters to produce 1000 baseline model fits (details in Supplementary Material, page 14). Model fits were used to produce the median and 95% credibility intervals (CrI) for all model projections. We validated our model fits against several data not used in the calibration process (Supplementary Table 4).

We used the best available evidence locally [23, 43, 44] or nationally [24] to explore the effects of disruptions in HCV- and HIV-related services due to the COVID-19 pandemic (Supplementary Table 5). We assumed that rates of HCV testing and treatment decreased by 59% over March-December 2020 compared to pre-March 2020 levels [23, 24]. The rate of HIV testing and proportion of MSM initiating HIV treatment also decreased by 31% over March-June 2020 [44] and PrEP initiation by 35% over March 2020 to March 2021, both compared to pre-March 2020 levels [43].

### Model Analyses

We evaluated the impact of ongoing and future HCV testing and treatment uptake on HCV transmission by estimating the relative reduction in HCV incidence over 2015–2022 or 2015–2030, and estimated the year when the relative reduction was 80% since 2015 (WHO target). Due to limited data on HCV and HIV service recovery following the COVID-19 pandemic, we modelled 3 possible recovery scenarios, and an additional scenario with increased high-coverage needle and syringe program (HCNSP) use:

- Status quo (SQ): No recovery. HCV and HIV testing and treatment and PrEP use following COVID-19 pandemic remain at pandemic levels.
- Scenario 1: Slow recovery. HCV and HIV testing and treatment and PrEP use return to prepandemic levels by end of 2025.
- Scenario 2: Rapid recovery. As scenario 1 but with recovery by end of 2022.
- Scenario 3: Rapid plus. As scenario 2 and with HCNSP scaled-up from 74% (NHBS-MSM 2017, unpublished) to 100% coverage of recent MSM-IDU over 2022–2026, and sustained thereafter, with HCNSP reducing HCV and HIV acquisition risks by 56% [45] and 42% [46], respectively.

The relative reduction in HCV incidence for these scenarios were compared to 2 counterfactual scenarios, one with no HCV testing and treatment over 2015–2030 and another with no scale-up in HCV testing and treatment in 2015. These were used to determine the percentage of the relative reduction in HCV incidence attributed to all HCV testing and treatment, or to the scale-up in HCV testing and treatment since 2015, respectively. We also evaluated the proportion of new HCV infections averted over 2023–2030 for scenarios SQ and 1–3, compared to an additional counterfactual where no testing or treatment occurred over 2023–2030. Lastly, we also projected what decrease in HCV incidence would have occurred if the COVID-19 pandemic had not occurred.

In sensitivity analyses, we explore the relative reduction in chronic HCV prevalence, as it could be a reliable alternative to validating HCV elimination [47].

### Uncertainty Analyses

A linear regression analysis of covariance (ANCOVA) was done to determine which parameter uncertainties contribute most to variability in the relative reduction in HCV incidence over 2015–2030 for scenario SQ. The proportion of each model outcome's sum-of-squares contributed by each parameter was calculated to estimate the importance of individual parameters to overall uncertainty. All simulations were performed using Matlab R2020a.

## RESULTS

**Figure 2.**
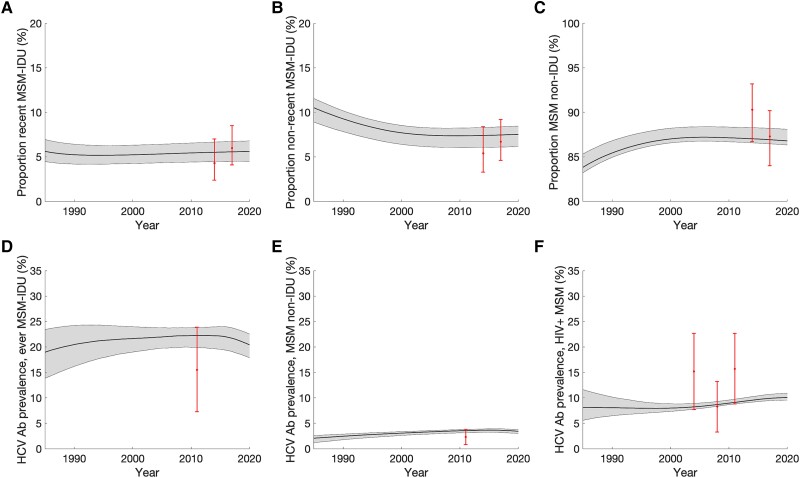
Model fit to calibration data on the proportion of MSM who injected recently, non-recently, and never (*A*–*C*) and on HCV Ab prevalence among ever MSM-IDU, MSM non-IDU and MSM with HIV (*D*–*F*). Black lines represent the median model projections and the shaded area represents the 95% credible intervals. Calibration data points with their 95% confidence intervals are indicated. Abbreviations: Ab, antibody; HCV, hepatitis C virus; HIV, human immunodeficiency virus; IDU, injection drug use; MSM, men who have sex with men.

**Figure 3.**
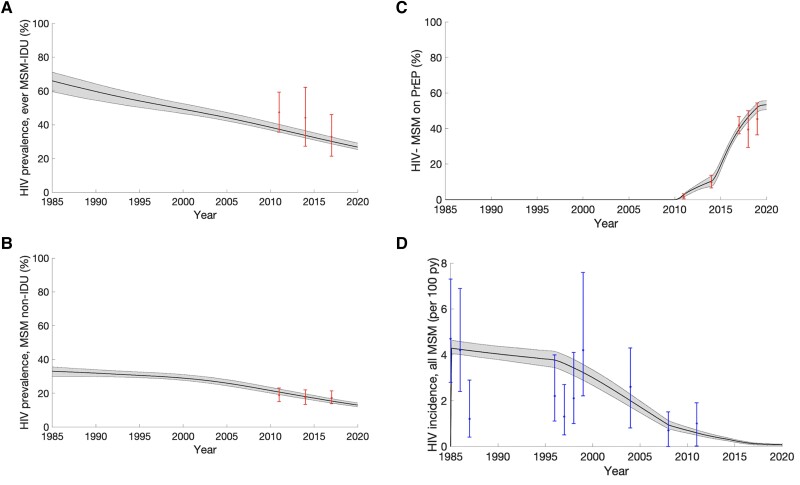
Model fit to calibration data on HIV prevalence among ever MSM-IDU and MSM non-IDU (*A* and *B*), and on the proportion of MSM on PrEP (*C*) and model fit to validation data on HIV incidence among all MSM (*D*). Black lines represent the median model projections and the shaded area represents the 95% credible intervals. Calibration data points with their 95% CIs are indicated in (*A*–*C*). Validation data with their 95% CIs are indicated in (*D*). Abbreviations: CI, confidence interval; HIV, human immunodeficiency virus; IDU, injection drug use; MSM, men who have sex with men.

**Figure 4.**
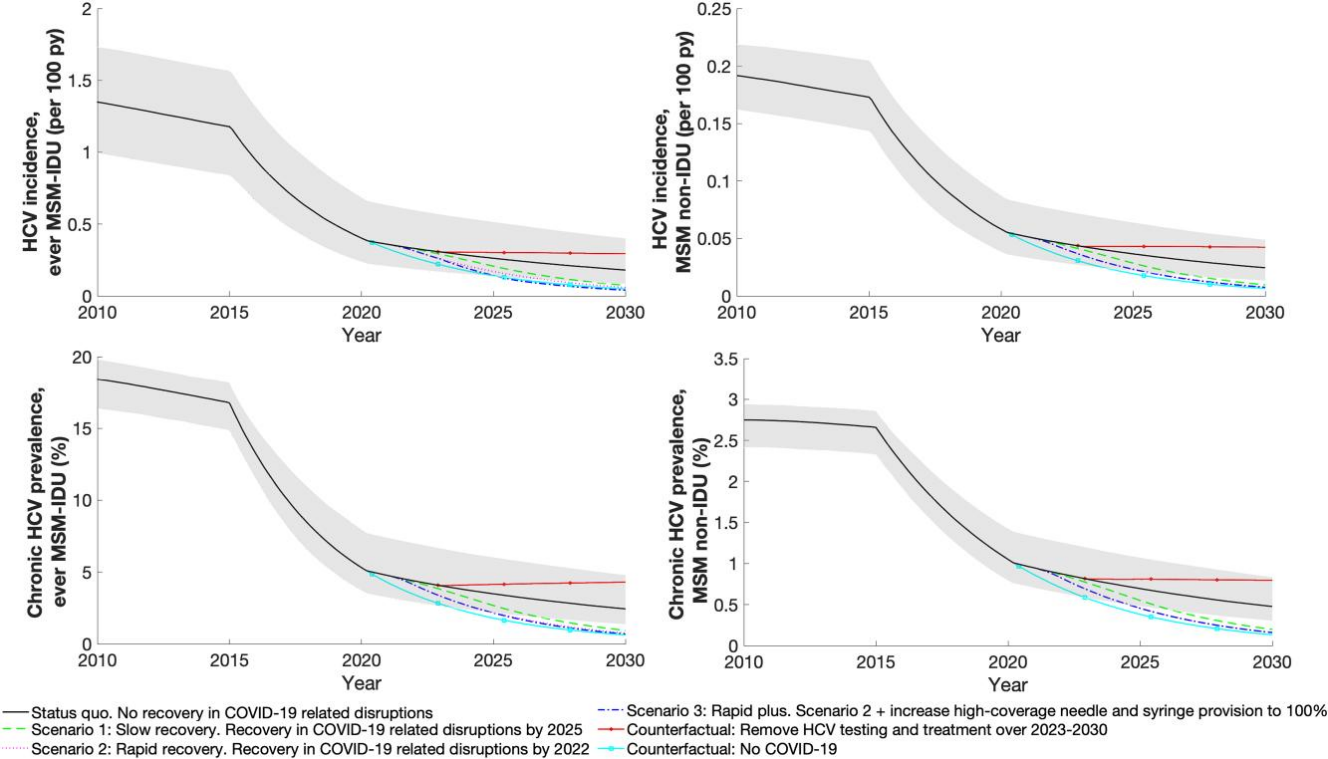
Projected HCV incidence and chronic HCV prevalence among MSM-IDU and MSM non-IDU, over 2010–2030. The shaded area reflects the 95% credible interval for the scenario in which we assume no rebound in COVID-19–related disruptions. All lines show median projection. Abbreviations: COVID-19, coronavirus disease 2019; HCV, hepatitis C virus; IDU, injection drug use; MSM, men who have sex with men; py, person year.

The model calibrated and validated well to data (Figure 2, Figure 3, and Supplementary Figures 1–7). It projects relatively stable proportions of MSM who inject drugs, with 5.1% (95% CrI, 4.3%–6.2%) and 7.8% (95% CrI, 6.4%–8.5%) being recent MSM-IDU and nonrecent MSM-IDU in 2022, respectively. Chronic HCV prevalence was relatively stable over time, with 16.8% (95% CrI, 14.8%–18.2%) and 2.7% (95% CrI, 2.3–2.9) prevalence among ever MSM-IDU and MSM non-IDU, respectively in 2022 (Figure 4). HIV prevalence is projected to have decreased over time and to be higher among ever MSM-IDU (24.5%; 95% CrI, 23.2%–26.8%) than MSM non-IDU (11.7%; 95% CrI, 10.8%–12.9%) in 2022.

In 2015, we estimated HCV incidence to be 1.2 per 100 person-years (95% CrI, 0.8–1.6) among ever MSM-IDU (Figure 4) and considerably greater among recent MSM-IDU (2.5; 95% CrI, 1.7–3.6) than nonrecent MSM-IDU (0.2; 95% CrI, 0.1–0.2). HCV incidence was the same among MSM non-IDU as nonrecent MSM-IDU. Among all MSM, 43.3% (95% CrI, 33.8%–51.8%) of incident HCV infections were attributed to IDU in 2022, increasing to 85.7% (95% CrI, 80.2%–89.5%) among ever MSM-IDU. For HIV, 2.8% (95% CrI, 1.8%–3.8%) and 20% (95% CrI, 14.5%–25.7%) of incident infections were attributed to IDU among all MSM and ever MSM-IDU, respectively.

### Impact of HCV Testing and Treatment Over 2015–2022

Following DAA scale-up in 2015, the estimated rates of HCV diagnosis and HCV treatment if diagnosed among ever MSM-IDU were 39.3/100 person-years (30.0–47.7) and 29.4/100 person-years (19.6–38.5; Supplementary Table 11), respectively. Assuming no recovery in HCV and HIV service disruptions following the COVID-19 pandemic (SQ), HCV incidence is projected to have decreased by 72.3% (95% CrI, 58.0%–79.7%) among ever MSM-IDU over 2015–2022, resulting in an estimated incidence of 0.3/100 person-years (95% CrI, 0.2–0.6; Supplementary Table 7) in 2022. Most of the decline in incidence was due to the impact of HCV testing and treatment over 2015–2022 (86.0%; 95% CrI, 80.5%–94.9%), and particularly the scale-up in these interventions since 2015 (65.4%; 95% CrI, 58.9%–72.7%; Supplementary Table 10).

The projected decreases in HCV incidence and chronic prevalence are similar in scenarios 1 and 2, which assume a recovery in HIV and HCV services following the COVID-19 pandemic (Supplementary Table 7). HCV incidence is estimated to have decreased to a similar extent over 2015–2022 among different MSM subgroups, as has chronic HCV prevalence (Supplementary Table 7).

### Impact of HCV Testing and Treatment Over 2015–2030

**Figure 5.**
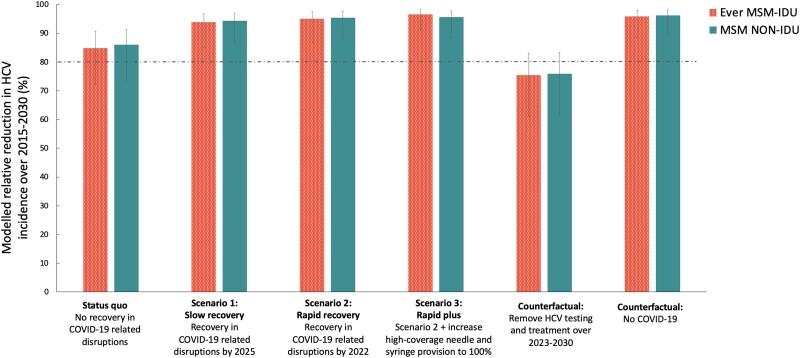
Modelled relative reduction in HCV incidence among ever MSM-IDU and MSM non-IDU in different scenarios over 2015–2030. Bars show the median projections, with whiskers showing the 95% credibility intervals. Abbreviations: COVID-19, coronavirus disease 2019; HCV, hepatitis C virus; IDU, injection drug use; MSM, men who have sex with men.

Assuming no recovery in HCV screening and treatment services following the COVID-19 pandemic (SQ), we project that HCV incidence among ever MSM-IDU will decrease by 84.9% (95% CrI, 72.3%–90.8%) over 2015–2030 (Figure 5 and Supplementary Table 8). Most of this decline in HCV incidence is due to the impact of HCV testing and treatment over this period (75.8%; 95% CrI, 66.7%–89.5%) and their scale-up since 2015 (54.1%; 95% CrI, 46.9%–64.6%; Supplementary Table 10). The WHO target for decreasing HCV incidence by 80% is projected to occur in 2026 for all MSM subgroups, but with wide uncertainty (95% CrI, 2022–2037 for ever MSM-IDU; Supplementary Figure 8).

If COVID-19–related service disruptions recovered by the end of 2025 (scenario 1/slow recovery) or 2022 (scenario 2/rapid recovery), then HCV incidence is projected to decrease by 93.9% (95% CrI, 84.9%–96.8%) or 95.0% (95% CrI, 86.9%–97.5%), respectively, over 2015–2030 among ever MSM-IDU (Figure 5 and Supplementary Table 8). In these scenarios, the HCV incidence target for elimination could be achieved slightly earlier (2024 [95% CrI, 2022–2028] and 2023 [95% CrI, 2022–2027], respectively). Also increasing HCNSP to 100% among recent MSM-IDU (scenario 2/rapid plus) has little additional impact.

In contrast, if HCV testing and treatment ceased over 2023–2030, HCV incidence starts to increase and only a 75.5% decrease (95% CrI, 61.0%–83.2%) occurs over 2015–2030 among ever MSM-IDU, with only 20% of model runs projecting an 80% decrease in incidence by 2030 (Figure 5 and Supplementary Table 8). Lastly, if the COVID-19 pandemic had not occurred, then incidence would have decreased by 95.9% (95% CrI, 88.5%–98.0%) over 2015–2030, achieving an 80% decrease in HCV incidence in 2023 (95% CrI, 2021–2026).

For all scenarios, results are similar for all MSM groups (Figure 5 and Supplementary Table 8). Chronic HCV prevalence is projected to decline to a similar extent to HCV incidence (Supplementary Table 8).

### Proportion of HCV Infections Averted Over 2023–2030

Compared to a counterfactual in which no HCV testing and treatment occurs over 2023–2030, continuing these services while assuming no recovery in services following the COVID-19 pandemic (SQ) would avert 20% (95% CrI, 14%–24%) of incident HCV cases over 2023–2030 among ever MSM-IDU (Supplementary Figure 9 and Supplementary Table 12). Conversely, if some recovery in COVID-19 services occurs (scenarios 1–3), then between 44% and 61% of new HCV infections would be averted (Supplementary Figure 9 and Supplementary Table 12). Similar proportions of cases would be averted for MSM non-IDU.

### Uncertainty Analyses

Our ANCOVA analyses indicate that variability in the relative reduction in HCV incidence achieved over 2015–2030 is mostly (74.1%) due to uncertainty in the scale-up of HCV treatment in 2015 (Supplementary Table 13).

## DISCUSSION

Our model suggests that, although IDU is only reported by a minority of MSM (13%), it accounts for nearly half (43%) of all incident HCV cases in this population and only 2.8% of HIV infections. We also found that HCV incidence has already decreased considerably (approximately 70%) over 2015–2022 among ever MSM-IDU in San Francisco, with this decline being largely (approximately 86%) attributed to the high levels of testing and treatment over this period. Trajectories of progress towards the WHO HCV incidence target seem modestly influenced by how fast disruptions in services recover following the COVID-19 pandemic, with most scenarios and projections suggesting an 80% reduction in HCV incidence over 2015–2030. Despite considerably higher HCV burden among ever MSM-IDU compared to MSM non-IDU, we project similar trajectories of change in HCV incidence for both. Going forward, it is essential that existing HCV interventions are sustained, as removing them would cause an increase in HCV incidence and prevent us from achieving the WHO target.

To our knowledge, this study is the first to consider IDU as a driver of HCV transmission among MSM in HCV transmission models, and to evaluate whether HCV testing and treatment services are reaching this high-risk group. It is also the first MSM-focused model of the HCV epidemic in the United States. We note several limitations. First, the independent contribution of IDU, relative to sexual risk, in driving HCV transmission among MSM is difficult to quantify and could have been misestimated in our study, even though it was informed by detailed local epidemiological data. IDU and sexual risk practices are self-reported, thus prone to misclassification due to recall errors and because they represent sensitive information. Furthermore, while we did not find evidence of a difference in sexual risk practices among MSM in our study by IDU status (Supplementary Material, page 3), several studies have suggested that these practices are often correlated [48], adding to the difficulty of isolating the independent contribution of each one. Second, we did not have data on HCV incidence; however, our projections align with HCV incidence estimates among MSM from other US settings [2]. Additionally, existing HCV treatment data among MSM in San Francisco precluded an assessment of potential differences by injecting and HIV-status. Although we utilized numerous established data sources among MSM to parametrize and calibrate our model to the local context, expanding data collection to include a systematic assessment of HCV incidence and treatment would strengthen projection modelling and programmatic response. Given that our projections are of the present, they can and should be tested through empirical HCV incidence data. Third, injection-related outcomes, such as injecting cessation and relapse and injection-related mortality, have been poorly characterized among MSM-IDU, which limited the data available for parameterizing our models. Fourth, it is unclear how HCV and HIV services have recovered following the COVID-19 pandemic. However, it is encouraging that the WHO HCV incidence target will likely be achieved even if there is no recovery.

Fifth, our model does not capture new HCV infections among MSM in San Francisco acquired through contacts with other populations. We did not model partnerships with people who inject drugs (PWID) due to evidence of limited interaction with this population [7]. Recent research led by our group suggested different sociodemographic characteristics and injecting and sexual risk behaviors between MSM-IDU (ie, men reached through affiliation with MSM) and men reached through affiliation with PWID, whether they engage or not in male-to-male sex (PWID-MSM and PWID non-MSM, respectively) [7]. International evidence using phylogenetic analyses also suggests that the HCV epidemics among MSM and PWID are distinct [49], though similar research is needed in San Francisco. We also did not model partnerships with MSM outside of San Francisco because of limited data. If HCV acquisition through unmodelled populations is significant, then we could be overestimating the impact of HCV testing and treatment on HCV incidence. Despite this limitation, our model represents an improvement on prior studies among MSM that only focused on MSM with HIV [11–15]. To our knowledge, only modelling done by our group has previously included MSM without HIV [16], despite evidence of high HCV incidence in this group [2], suggestive of shared transmission networks with MSM with HIV.

Our findings indicating a pronounced decrease in HCV incidence among ever MSM-IDU and MSM non-IDU likely reflect San Francisco's multilayered efforts to increase HCV service access for high-risk populations through integration of services. San Francisco has long been a leader in the prevention and treatment of HIV for MSM, and several HIV-focused programs have added HCV testing and treatment into routine sexual health care. It developed the first city-focused strategic plan to eliminate HCV [17] and an HCV “microelimination plan” for people with HIV. While empirical data supporting the benefit of HCV treatment-as-prevention are only starting to emerge [50], other modelling studies have shown that marked reductions in HCV incidence among MSM can be achieved [11, 12, 14–16] through scaling-up HCV testing and DAA treatment.

We note that results are unlikely to be representative of the wider MSM-IDU and MSM communities in the United States. Few US states have implemented HCV elimination plans and MSM elsewhere often have lower access to services compared to those based in San Francisco. Furthermore, additional research is needed to assess whether other populations with overlapping risk behaviors are adequately served by HCV prevention and treatment services. We recently reported that, compared to MSM-IDU, PWID-MSM in San Francisco present greater socioeconomic disadvantage, have higher HCV prevalence, and lower engagement in services [7], suggesting that the impact of current HCV testing and treatment programs could be less favorable in this group.

In conclusion, despite a considerably higher HCV burden among ever MSM-IDU relative to MSM non-IDU in San Francisco and recent reductions in HCV testing and treatment due to the COVID-19 pandemic, our model suggests that the WHO HCV incidence target will be achieved in both populations. This finding can be attributed to the high intensity of HCV testing and treatment among MSM in this city, prior to COVID-19 disruptions, and illustrates what can be achieved by creating a robust HCV elimination program that prioritizes vulnerable populations.

## Supplementary Data

Supplementary materials are available at *The Journal of Infectious Diseases* online. Consisting of data provided by the authors to benefit the reader, the posted materials are not copyedited and are the sole responsibility of the authors, so questions or comments should be addressed to the corresponding author.

## Supplementary Material

jiad169_Supplementary_Data

## Notes

***Author contributions.*** A. A., J. S., S. N. F., H. F., P. V., and M. D. M. conceptualized the model and aims. A. A. developed the model, reviewed the literature, performed analyses on unpublished data for model parametrization, and wrote a first draft of the manuscript. J. S. and P. V. oversaw the modelling. S. N. F., J. H., P. R. III, W. M., E. W., and M. D. M. collected or contributed data for modelling and provided feedback on model development. All authors contributed to data interpretation, writing the manuscript, and approved the final version.

***Disclaimer***. The funding sources had no role in the design, collection, analysis, and interpretation of data; writing of the report; or the decision to submit the report for publication.

***Financial support*.** This work was supported by the Canadian Institute of Health Research, Fonds de Recherche du Québec-Santé, and Canadian Network on Hepatitis C (postdoctoral fellowships to A. A.); the National Institute of Health Research Health Protection Research Unit in Behavioural Science and Evaluation at University of Bristol (to H. F. and P. V.); and the National Institute for Drug Abuse (grant numbers R01DA033679 and R21DA047902 to H. F. and P. V., and 1R21DA046809 to S. F., H. F., P. V., and M. M.).
