# Supplementary material for: Impact of HCV Testing and Treatment on HCV Transmission Among Men Who Have Sex With Men and Who Inject Drugs in San Francisco: A Modelling Analysis
Source: J Infect Dis. 2023 Jul 24;228(6):662–73. doi: 10.1093/infdis/jiad169 (PMC10503949; doi:10.1093/infdis/jiad169)
Supplement: jiad169_Supplementary_Data [file jiad169_supplementary_data.docx]

Table of Contents

[**1.0** **DETAILS ON KEY PARAMETERS** 3](#_Toc133083033)

[**1.1 SEXUAL PRACTICES BY INJECTING STATUS** 3](#_Toc133083034)

[**1.2 HCV TESTING** 3](#_Toc133083035)

[1.2.1 Differences in HCV testing by injecting and HIV infection statuses 3](#_Toc133083036)

[1.2.2 Rate of HCV testing among MSM non-IDU & HIV-negative not on PrEP in 2017 4](#_Toc133083037)

[1.2.3 Trends in HCV testing over time 4](#_Toc133083038)

[**1.3. RATE OF HCV TREATMENT INITIATION** 4](#_Toc133083039)

[**1.4. DIFFERENCES IN HIV TESTING BY INJECTING AND PREP STATUSES** 5](#_Toc133083040)

[**1.5. PROPORTION OF HIV POSITIVE MSM ON ART** 5](#_Toc133083041)

[1.5.1 Trends in proportion of MSM on ART over time 5](#_Toc133083042)

[1.5.2 Differences in the proportion of MSM on ART by injecting status 6](#_Toc133083043)

[**1.6. DIFFERENCES IN PREP INITIATION BY INJECTING STATUS** 6](#_Toc133083044)

[**2.0 MODEL EQUATIONS** 8](#_Toc133083045)

[**3.0 CALIBRATION DETAILS** 14](#_Toc133083046)

[**SUPPLEMENTARY TABLES** 15](#_Toc133083047)

[**Supplementary Table 1:** Data sources 15](#_Toc133083048)

[**Supplementary Table 2:** Studies among MSM illustrating the relationships between IDU and sexual risk behaviours with incident or prevalent HCV infection 16](#_Toc133083049)

[**Supplementary Table 3:** Prior ranges for parameters used in the model 17](#_Toc133083050)

[**Supplementary Table 4:** Data used to calibrate and validate the model 22](#_Toc133083051)

[**Supplementary Table 5:** Changes in HIV and HCV services due to the COVID-19 pandemic: data and assumptions 24](#_Toc133083052)

[**Supplementary Table 6:** Results**—**Modelled incidence and chronic prevalence in 2015 25](#_Toc133083053)

[**Supplementary Table 7:** Results**—**Modelled incidence and chronic prevalence in 2022 and relative reduction over 2015-2022, depending on the level of recovery in COVID-19 related service disruptions 26](#_Toc133083054)

[**Supplementary Table 8:** Results**—**Projected HCV incidence and chronic HCV prevalence in 2030 and relative change over 2015-2030 27](#_Toc133083055)

[**Supplementary Table 9:** Results**—**Year when HCV incidence is estimated to decrease by 80% compared to 2015 levels 28](#_Toc133083056)

[**Supplementary Table 10:** Results**—**Estimated contribution of HCV testing and treatment to the decline in HCV incidence among MSM 29](#_Toc133083057)

[**Supplementary Table 11:** Results – Modelled rates of HCV diagnosis and HCV treatment 30](#_Toc133083058)

[**Supplementary Table 12:** Results—Number and proportion of incident HCV cases averted over 2023-2030 compared to a scenario in which the standard of care is removed over 2023-2030 (scenario 4) 31](#_Toc133083059)

[**Supplementary Table 13:** Results**—**Posterior ranges for parameters included in the model 32](#_Toc133083060)

[**Supplementary Table 14:** Results**—**Uncertainty analyses 34](#_Toc133083061)

[**SUPPLEMENTARY FIGURES** 35](#_Toc133083062)

[**Supplementary Figure 1:** Model fit to calibration data for (A) MSM population size and (B) HIV-negative ever MSM-IDU 35](#_Toc133083063)

[**Supplementary Figure 2:** Model fit to calibration data on proportion of HCV-diagnosed MSM who were ever treated 36](#_Toc133083064)

[**Supplementary Figure 3:** Model fit to calibration data on proportion of HIV-positive MSM who are diagnosed 37](#_Toc133083065)

[**Supplementary Figure 4:** Model fit to validation data on proportion of MSM who injected in the previous year (recent MSM-IDU) 38](#_Toc133083066)

[**Supplementary Figure 5:** Model fit to validation data on proportion of MSM ever chronically infected with HCV who were ever diagnosed 39](#_Toc133083067)

[**Supplementary Figure 6:** Model fit to validation data on HCV Ab prevalence among (A) all MSM, (B) HIV+ ever MSM-IDU, (C) HIV+ MSM non-IDU and (D) HIV- MSM non-IDU 40](#_Toc133083068)

[**Supplementary Figure 7:** Model fit to validation data on HIV prevalence, all MSM 41](#_Toc133083069)

[**Supplementary Figure 8:** Projected year when HCV incidence decreases by 80% compared to 2015 levels among ever MSM-IDU and MSM non-IDU, for different scenarios* 42](#_Toc133083070)

[**Supplementary Figure 9:** Proportion of incident cases of HCV infection averted over 2023-2030 among ever MSM-IDU and MSM non-IDU for different scenarios, compared to a scenario in which there is no HCV testing and treatment over 2023-2030 43](#_Toc133083071)

# **DETAILS ON KEY PARAMETERS**

## **1.1 SEXUAL PRACTICES BY INJECTING STATUS**

Number of male sexual partners in the past 12 months, NHBS 2017 data:

|  | **Median (IQR)** | **P-value** |
| --- | --- | --- |
| Recent MSM-IDU (N=30) | 10 (6-20) | 0.37 |
| Non-recent MDM-IDU (N=32) | 6 (2-25) |  |
| MSM non-IDU (N=434) | 7 (2-20) |  |

Condom use at last anal sex, NHBS 2017 data:

|  | **n (%)** | **P-value** |
| --- | --- | --- |
| Ever MSM-IDU (N=39) | 7 (18%) | 0.44 |
| MSM non-IDU (N=242) | 57 (23.6%) |  |

**only applies to MSM who responded having had sex in the previous 12 months*

## **1.2 HCV TESTING**

### 1.2.1 Differences in HCV testing by injecting and HIV infection statuses

Data on HCV testing in the previous 12 months among MSM are only available in NHBS 2017. In this survey, the proportion of MSM* reporting testing for HCV in the past 12 months by injecting and HIV infection statues are:

|  | **HIV positive MSM** | **HIV negative MSM on PrEP** | **HIV negative MSM, not on PrEP** |
| --- | --- | --- | --- |
| **Ever MSM-IDU** | 9/15=60% | 10/18=55% | 9/21=43% |
| **MSM non-IDU** | 34/69=49% | 55/150=37% | 33/191=17% |

*Data were examined among self-reported HCV-negative MSM, as HCV-testing is not expected to have been done among HCV-positives

Other data in the US and testing recommendations suggest that MSM have high levels of HCV testing and people with a history of injection drug use, those who are on PrEP and those who are HIV positive may be more likely to be tested than other groups^[1, 2]^. Therefore, we used the "MSM non-IDU and HIV-negative not on PrEP" as reference category to estimate the relative risks (and 95% CI) of HCV testing in the previous 12 months for all other groups:

|  | **HIV positive MSM** | **HIV negative MSM on PrEP** | **HIV negative MSM not on PrEP** |
| --- | --- | --- | --- |
| **Ever MSM-IDU** | 3.5 (2.1 – 5.8) | 3.2 (1.9 – 5.4) | 2.5 (1.4 – 4.4) |
| **MSM non-IDU** | 2.9 (1.9 – 4.2) | 2.1 (1.5 – 3.1) | Ref. |

Given that the relative risks for HCV testing for all five groups are (i) higher compared to the reference category, (ii) do not differ considerably among each other and (iii) the 95% CI are imprecise due to small numbers in some groups, we pooled data from "ever MSM-IDU, MSM who are HIV negative MSM on PrEP and MSM who are HIV positive" into a single group assumed to all have the same relative risk of HCV testing compared to "MSM non-IDU, HIV-negative not on PrEP", as follows:

|  | **Tested for HCV, past 12 months** | **Not tested for HCV, past 12 months** | **Relative risk** |
| --- | --- | --- | --- |
| **MSM non-IDU and HIV negative not on PrEP** | 33 | 158 | Ref. |
| **Ever MSM-IDU and/or HIV negative on PrEP or HIV positive** | 117 | 155 | 2.5 (1.8 – 3.5) |

### 1.2.2 Rate of HCV testing among MSM non-IDU & HIV-negative not on PrEP in 2017

Using data from NHBS 2017 on proportion tested for HCV in the previous 12 months among MSM non-IDU and HIV negative not on PrEP, we fitted a small model to estimate the yearly rate of HCV testing, as follows:


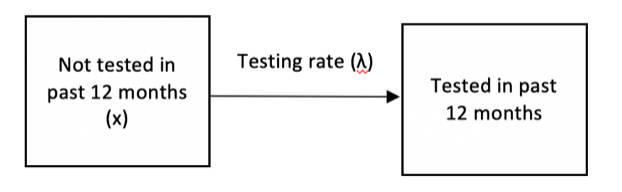


Assuming an exponential decay function:

dx/dt = - λ *t 🡪 x_1_ = A_0_ * $e^{-\lambda*1}$

A_0_ = 1

Proportion tested, previous 12 months= 17.3% (95% CI: 12.2% - 23.4%); normal distribution (mean: 17.3%, SE: 0.03; truncated at the 95% CI)

x_1_ = 1- Proportion tested p12m

λ_1_ = -ln (x_1_)

### 1.2.3 Trends in HCV testing over time

To explore trends over time in HCV testing, we explored trends in the proportion of MSM reporting ever being tested in NHBS (2011, 2014 and 2017) and SFAF MSM street-intercept study (2017, 2018, 2019) [unpublished data]:

| **Data source and year** | **Proportion of MSM ever tested for HCV (95% CI)** | **Relative Risk (95% CI)** |
| --- | --- | --- |
| NHBS 2011 | 79.1 (75.1 – 82.7) | Ref |
| NHBS 2014 | 81.1 (76.6 – 85.1) | 1.03 (0.96 - 1.1) |
| NHBS 2017 | 83.6 (80.0 – 86.8) | 1.06 (0.99 - 1.12) |
| SFAF MSM street-intercept study 2017 | 89.1 (86.3 – 91.5) | 1.13 (1.07 - 1.19) |
| SFAF MSM street-intercept study 2018 | 85.8 (78.3 – 91.5) | 1.09 (1.00 - 1.18) |
| SFAF MSM street-intercept study 2019 | 86.2 (80.5 – 90.8) | 1.10 (1.01 - 1.17) |

To explore trends in HCV testing before 2011, data from two published studies using earlier rounds of NHBS (2004-2011) suggest that, among HIV positive MSM, proportion of HCV testing was high:

| **Data source and year** | **Proportion of HIV positive MSM who tested HCV Ab positive and were aware of having been infected with HCV (95% CI)** |
| --- | --- |
| NHBS 2004^[3]^ | 71.4% (41.9% - 91.6%) |
| NHBS 2008^[3]^ | 90.0% (55.5% - 99.8%) |
| NHBS 2011^[4]^ | 70.6% (44.0% - 89.7%) |

Based on these different data, we assumed that HCV testing among MSM was initiated in 1999-2001, increased linearly until 2017, after which it remained constant.

## **1.3. RATE OF HCV TREATMENT INITIATION**

Pre-DAAs: In a study conducted among HIV-infected patients under care at the University of California, San Francisco, 15.7% (95% CI: 12.8% - 18.9%) of patients with chronic HCV were treated over 2008-2012^[5]^. Several studies conducted in the US^[6, 7]^, including a nationwide study^[6]^, indicate that during the interferon-era, HCV treatment was largely similar in HIV-positive and HIV-negative individuals. Based on these studies, we estimated the yearly rate of HCV treatment initiation in 2012 among all MSM, irrespective of HIV status, by fitting a small model as follows:


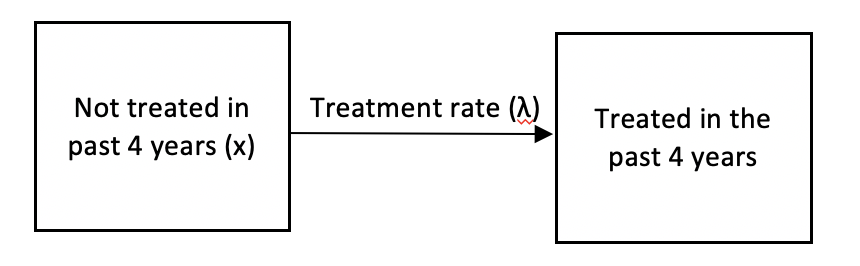


Assuming an exponential decay:

dx/dt = - λ *t 🡪 x_4_ = A_0_ * $e^{-4\lambda}$

A_0_ = 1

X_4_ = 1- Proportion treated/previous four years

λ = -ln(x_4_)/4

We assumed HCV treatment to have started in 2002-2004^[8]^. Thereafter, we assumed the rate of HCV treatment initiation to have scaled-up linearly to reproduce the 2012 HCV treatment initiate rate derived from the aforementioned study. Between 2012 and 2014, we assumed levels to have remained stable.

Post-DAAs: Data from NHBS and SFAF MSM street-intercept study indicate that the proportion of MSM who were ever treated for HCV among those ever diagnosed with chronic HCV is:

| **Data source and year** | **Estimate (95% CI)** |
| --- | --- |
| NHBS 2017 (unpublished) | 61.1% (95%: 35.8% - 82.7%) |
| SFAF MSM street-intercept study 2018 (unpublished) | 75% (34.9% - 96.8%) |
| SFAF MSM street-intercept study 2019 (unpublished) | 57.1% (18.4% - 90.1%) |

Given large uncertainty in the estimates, we pooled these estimates, and assumed the pooled estimate to reflect 2018 levels: 63.6% (45.1% - 79.6%).

US-data suggests that HCV treatment was scaled-up in 2015 following the introduction of DAAs^[9]^. Therefore, we assumed that the rate of HCV treatment initiation was scaled-up in 2015 and remained stable afterwards. The level of scale-up was allowed to vary between 1-10 (uniform distribution) to reproduce the aforementioned 2018 levels of MSM with HCV infection ever reported to have been treated.

## **1.4. DIFFERENCES IN HIV TESTING BY INJECTING AND PREP STATUSES**

In NHBS 2017, 81.7% (95% CI: (77.5% - 85.4%)) of HIV negative MSM reported being tested for HIV in the previous year (unpublished). The proportion of participants reporting HIV testing by injecting and PrEP statuses is:

| **Group** | **Estimate (95% CI)** | **P-value** |
| --- | --- | --- |
| MSM non-IDU | 81.3% (76.8% - 85.3%) | 0.72 |
| Recent MSM-IDU | 80.0% (56.3% - 94.3%) |  |
| Non-recent MSM-IDU | 90.0% (68.3% - 98.8%) |  |
| HIV negative MSM not on PrEP | 67.6% (60.8% - 73.9%) | <0.01 |
| HIV negative MSM on PrEP | 98.3% (95.2% - 99.7%) |  |

Based on the above, we assume that the rate of testing for HIV does not vary by injecting status. Given that nearly all MSM on PrEP were tested for HIV in the previous year, in line with CDC guidelines recommending that people on PrEP be tested frequently (i.e., every three months)^[10]^, we assumed that once infected, this group is diagnosed right away.

## **1.5. PROPORTION OF HIV POSITIVE MSM ON ART**

### 1.5.1 Trends in proportion of MSM on ART over time

The proportion of HIV-positive and diagnosed MSM who are on ART, based on NHBS and SFAF MSM street-intercept study data:

| **Data source and year** | **Proportion of HIV positive MSM on ART** |
| --- | --- |
| NHBS 2008^[11]^ | 79.3% (70.6% - 87.3%) |
| NHBS 2011^[11]^ | 88.2% (82.1% - 94.3%) |
| NHBS 2014 (unpublished) | 94.3% (86.0% - 98.4%) |
| NHBS 2017 (unpublished) | 95.7% (89.4% - 98.8%) |
| SFAF MSM street-intercept study 2017 (unpublished) | 91.9% (84.0% - 96.7%) |
| SFAF MSM street-intercept study 2018 (unpublished) | 87.5% (61.7% - 98.5%) |
| SFAF MSM street-intercept study 2019 (unpublished) | 85.2% (66.3% - 95.8%) |

ART was assumed to have started in 1996^[12]^. Based on the data above, we assumed that the proportion of HIV positive and diagnosed MSM on ART increased linearly from 1996 to reproduce 2008 and 2017 levels and remain stable afterwards. In 2017, we used a weighted average based on NHBS and SFAF MSM street-intercept study data (93.8% (86.7 – 97.8)).

### 1.5.2 Differences in the proportion of MSM on ART by injecting status

The proportion of HIV-positive and diagnosed MSM on ART, stratified by IDU status, based on NHBS data:

| **Injection group** | **Estimate (95% CI)** | **Data source and year** | **P-value*** |
| --- | --- | --- | --- |
| MSM non-IDU | 97.1% (89.9% - 99.7%) | NHBS 2017 (unpublished) | 0.07 |
| Recent MSM-IDU | 77.8% (40.0% - 97.2%) | NHBS 2017 (unpublished) |  |
| Non-recent MSM-IDU | 100% (71.5% 100%)** | NHBS 2017 (unpublished) |  |
| MSM non-IDU | 96.2% (86.8% - 99.5%) | NHBS 2014 (unpublished) | 0.12 |
| Recent MSM-IDU | 100% (59.0% - 100%)** | NHBS 2014 (unpublished) |  |
| Non-recent MSM-IDU | 75.0% (34.9% - 96.8%) | NHBS 2014 (unpublished) |  |
| MSM non-IDU | 91.2% (81.8% - 96.7%) | NHBS 2011 (unpublished) | 0.03 |
| Recent MSM-IDU | 63.6% (30.8% - 89.1%) | NHBS 2011 (unpublished) |  |
| Non-recent MSM-IDU | 95.5% (77.2% - 99.9%) | NHBS 2011 (unpublished) |  |

* P-value based on comparing estimates across all three injecting groups in each NHBS survey

** one-sided 95% CI

Given that the estimates are unstable and fluctuate a lot across recent and non-recent MSM-IDU over time, we used one single estimate for the proportion of HIV-positive and diagnosed MSM on ART.

## **1.6. DIFFERENCES IN PREP INITIATION BY INJECTING STATUS**

In NHBS 2017, the proportion of HIV negative MSM who indicated receiving PrEP in the previous year was 44.0% (39.0% - 49.1%) (unpublished). The proportion of MSM who reported PrEP in the previous year by injecting status is:

| **Injection group** | **Estimate (95% CI)** | **P-value** |
| --- | --- | --- |
| HIV-negative MSM non-IDU | 45.4% (40.1% - 50.8%) | 0.98 |
| HIV-negative recent MSM-IDU | 45.0% (23.1% - 68.5%) |  |
| HIV-negative non-recent MSM-IDU | 47.6% (25.7% - 70.2%) |  |

Based on the above, we assume that the rate of PrEP initiation does not vary by injecting status.

We have used the measure "PrEP use in the previous year" to calibrate the rate of PrEP initiation because this is the metric available over time in NHBS and SFAF MSM street-intercept study. Although this could theoretically over-estimate the proportion of MSM on PrEP currently or recently, we found no difference between these two metrics in NHBS 2017 (44.0% (95% CI: 39.0% - 49.1%) and 40.6% (95% CI: 35.7% - 45.7%) MSM reported using PrEP in the previous year and previous month, respectively (unpublished)).

# **2.0 MODEL EQUATIONS**

We denote the proportion of the population in each state by using compartments, $X_{ijk}$, where:

- **Subscript** $\boldsymbol{i}$ denotes the HCV-status (*i* = 1, HCV susceptible (Ab- RNA-); *i* = 2, spontaneously cleared and HCV susceptible (Ab+ RNA-); *i* = 3 chronic undiagnosed HCV (Ab+ RNA+); *i* = 4 Chronic diagnosed HCV (Ab+ RNA+); *i* = 5, on treatment for HCV (Ab+ RNA-); *i* = 6, failed HCV treatment (Ab+ RNA+); *i* = 7, cured through treatment and HCV susceptible (Ab+ RNA-); *i* = 8, chronic undiagnosed HCV (Ab+ RNA+); *i* = 9, chronic diagnosed HCV (Ab+ RNA+))
- **Subscript** $\boldsymbol{j}$ denotes the HIV-status (*j* = 1, HIV susceptible and not on PrEP; *j*=2 HIV susceptible and on PrEP; *j* = 3 Undiagnosed HIV; *j* = 4 Diagnosed HIV)
- **Subscript**$\boldsymbol{k}$ denotes the injecting status (*k* = 1, MSM non-IDU; *k* = 2, recent MSM-IDU; *k* = 3, non-recent MSM-IDU). We define MSM who have injected in the previous year as recent MSM-IDU, those who have injected previously but not in the past year as non-recent MSM-IDU, and MSM with no history of injecting as MSM non-IDU.

**Symbols and definitions:**

| $\beta_{HIV}^{idu}$ | injecting-related HIV transmission rate |
| --- | --- |
| $\beta_{HCV}^{idu}$ | injecting-related HCV transmission rate |
| $\beta_{HIV}^{sex}$ | sexual-related HIV transmission rate |
| $\beta_{HCV}^{sex}$ | sexual-related HCV transmission rate |
| $\psi$ | increased HCV infectivity for MSM who are HIV-infected, regardless of whether they are or not they are on ART |
| $(t)$ | proportion of HIV-positive and diagnosed MSM on ART |
| ${}_{1}$ | Proportion of HIV positive and diagnosed MSM who are on ART in 2008 |
| ${}_{2}$ | Proportion of HIV positive and diagnosed MSM who are on ART in 2017 |
|  | relative risk reflecting reduced injecting- and sexual-related HIV infectivity among those on ART |
| ${}_{1}$ | reduced susceptibility to injecting-related HIV acquisition among MSM on PrEP |
| ${}_{2}$ | reduced susceptibility to sexually-related HIV acquisition among MSM on PrEP |
| $\lambda^{idu}$ | HCV force of infection due to injecting transmission among recent MSM-IDU |
| $\lambda^{sex}$ | HCV force of infection due to sexual transmission among all MSM |
| $\Lambda_{k}$ | Force of infection for HCV |
| ${\sigma_{1}}_{idu}$ | HIV force of infection due to injecting transmission among recent MSM-IDU not on PrEP |
| ${\sigma_{2}}_{idu}$ | HIV force of infection due to injecting transmission among recent MSM-IDU on PrEP |
| ${\sigma_{1}}_{sex}$ | HIV force of infection due to sexual transmission among all MSM not on PrEP |
| ${\sigma_{2}}_{sex}$ | HIV force of infection due to injecting transmission all among MSM on PrEP |
| $S_{jk}$ | Force of infection for HIV |
| $\Phi_{j}$ | Proportion of individuals who spontaneously clear HCV infection |
| $\phi_{1}$ | Proportion of individuals who spontaneously clear HCV infection among HIV-negative MSM |
| $\phi_{2}$ | Proportion of individuals who spontaneously clear HCV infection among HIV-positive MSM |
| $D_{jk}(t)$ | Rate of HCV diagnosis |
| $D_{start}$ | Year when HCV testing/diagnosis for HCV started |
| $\delta_{prop}$ | Proportion of MSM non-IDU, who were HIV-negative and not on PrEP and reported HCV testing in the past year |
| $\delta\left( t \right)$ | Rate of HCV diagnosis among MSM non-IDU, who were HIV-negative and not on PrEP |
| $RR_{dHCV}$ | Relative risk of HCV diagnosis among MSM who were ever MSM-IDU or HIV negative MSM on PrEP or HIV positive MSM compared to MSM non-IDU who were HIV-negative and not on PrEP |
| $M_{j}$ | Rate of HCV-related mortality |
| $RR_{m_{HCV}}$ | Relative risk of HCV-related mortality among MSM who are co-infected with HIV |
| $\mu_{HCV}$ | HCV-related mortality among MSM who are not infected with HIV |
| $T_{x}\left( t \right)$ | Rate of HCV treatment |
| ${T_{x}}_{start}$ | Year interferon-based HCV treatment started |
| ${T_{x}}_{Pre-DAA}$ | Proportion of diagnosed MSM treated pre-DAAs over a 4-year period |
| ${RR}_{Post-DAA}$ | Relative risk reflecting the increase in rate of HCV treatment in 2015 due to DAA scale-up relative to previous years |
| $\gamma(t)$ | Duration of HCV treatment |
| $\gamma_{1}$ | Duration of treatment with interferon |
| $\gamma_{2}$ | Duration of treatment with DAAs |
| ${SVR}_{j}$ | Proportion of HCV treatments that result in SVR |
| $\tau_{1}$ | Proportion of HCV treatments that result in SVR among HIV-negative MSM |
| $\tau_{2}$ | Proportion of HCV treatments that result in SVR among HIV-positive MSM |
| ${eff\_preDAAs}_{HIVn}$ | Proportion of HCV treatments that result in SVR during the interferon era (2004-2014) among HIV-negative MSM |
| ${eff\_preDAAs}_{HIVp}$ | Proportion of HCV treatments that result in SVR during the PEG-IFN era (2004-2014) among HIV-positive MSM |
| $eff\_postDAAs$ | Proportion of HCV treatments that result in SVR during the DAA era (2015-onward) irrespective of HIV status |
| $PREP\left( t \right)$ | Rate of PrEP initiation |
| $\pi_{1}$ | Rate of PrEP initiation in 2011 |
| $\pi_{1}$ + $\pi_{2}$ | Rate of PrEP initiation in 2014 |
| $\pi_{1}+$ $\pi_{2}+$ $\pi_{3}$ | Rate of PrEP initiation in 2019 |
| $\eta$ | Rate of PrEP cessation |
| $D_{HIV}(t)$ | Rate of HIV diagnosis |
| $\varepsilon$ | Rate of HIV testing in 2017 |
| $\alpha$ | Rate at which MSM initiate injection drug use |
| $\mu_{1}$ | Rate of aging out |
| $\mu_{3}$ | Rate of background mortality |
| $\upsilon$ | Rate at which MSM stop injection drug use |
| $\mu_{2}$ | Injection drug use-related mortality |
| $\mu_{4}$ | Rate of HIV-related mortality among MSM who are not on ART |
| $RR_{ART}$ | Relative risk of HIV-related mortality among MSM who are on ART vs MSM who are not on ART |

The ordinary differential equation models can be written as:

$\frac{\partial X_{ijk}}{\partial t}=A_{ijk}+B_{ijk}+C_{ijk}$, where:

$A_{ijk}$ represents movement due to the HCV transmission, diagnosis and treatment

$B_{ijk}$ represents movement due to HIV transmission, diagnosis and treatment

$C_{ijk}$ represents movement due to injecting drug use, inflow and background mortality

Each of these terms is described in more detail below.

**For movement due to HCV:**

$$A_{1jk} =-\Lambda_{k} X_{1jk}$$

$$A_{2jk} =\Phi_{j} \Lambda_{k}X_{1jk}-(1-\Phi_{j})\Lambda_{k}X_{2jk}$$

$$A_{3jk} =\left( 1-\Phi_{j} \right)\Lambda_{k}X_{1jk}+\left( 1-\Phi_{j} \right)\Lambda_{k}X_{2jk}-{(D}_{jk}\left( t \right)+M_{j})X_{3jk}$$

$$A_{4jk} =D_{jk}{\left( t \right) X}_{3jk}-{(T}_{x}\left( t \right)+M_{j})X_{4jk}$$

$$A_{5jk} =T_{x}\left( t \right){(X}_{4jk}+X_{6jk}+X_{9jk})-\gamma\left( t \right)X_{5jk}$$

$$A_{6jk} =\gamma(t)\left( 1-SVR_{j} \right)X_{5jk}-T_{x}\left( t \right)X_{6jk}-M_{j}X_{6jk}$$

$$A_{7jk} =\gamma(t) SVR_{j}X_{5jk}-\left( 1-\Phi_{j} \right)\Lambda_{k}X_{7jk}$$

$$A_{8jk} =\left( 1-\Phi_{j} \right)\Lambda_{k}X_{7jk}-{(D}_{jk}\left( t \right)+M_{j})X_{8jk}$$

$$A_{9jk} =D_{jk}\left( t \right)X_{8jk}-\left( T_{x}\left( t \right)+M_{j} \right)X_{9jk}$$

where:

- $\Lambda_{k}$ is the force of infection for HCV among MSM in injecting state *k* (see section below)
- $\Phi_{j}$ is the proportion of individuals who spontaneously clear HCV infection, which was assumed to vary as a function of HIV status, where

$$\Phi_{j}=\left\{ \begin{aligned} \phi_{1}, j=1,2 \\ \phi_{2}, j=3,4 \end{aligned} \right.$$

- $D_{jk}(t)$ is the rate of HCV diagnosis for an individual in HIV infection state *j* and injecting state *k* (see section below)
- $M_{j}$ is the rate of HCV-related mortality for an individual in HIV infection state *j* (see section below)
- $T_{x}\left( t \right)$ is the HCV treatment rate (see section below)
- $\gamma(t)$ is the duration of treatment, where

$$\gamma(t)=\left\{ \begin{matrix} 0, & t<{T_{x}}_{start} \\ \gamma_{1}, & {T_{x}}_{start}\leq t\leq2015 \\ \gamma_{2}, & 2015<t \end{matrix} \right.$$

- ${SVR}_{j}$ is the SVR rate which depends on HIV infection state *j* (see section below)

HCV force of infection ($\Lambda_{k}$)

We assume that the injecting-related HCV infectivity for MSM who are HIV-positive is increased by a factor $\psi$ compared to MSM who are HIV-negative, regardless of whether they are on ART. We define the HCV force of infection due to injecting transmission among recent MSM-IDU as:

$$\lambda^{idu}=\beta_{HCV}^{idu}\frac{\Sigma_{i=3,4,6,8,9}\Sigma_{j=1,2} X_{i,j,2}+ \psi\Sigma_{i=3,4,6,8,9}\Sigma_{j=3,4} X_{i,j,2}}{\Sigma_{ij}X_{i,j,2}}$$

where $\beta_{HCV}^{idu}$ is the injecting-related HCV transmission rate

We assume that the sexually-related HCV infectivity for MSM who are HIV-positive is increased by a factor $\psi$ compared to MSM who are HIV-negative, regardless of whether or not they are on ART. We define the HCV force of infection due to sexual transmission among all MSM as:

$$\lambda^{sex}=\beta_{HCV}^{sex}\frac{\Sigma_{i=3,4,6,8,9}\Sigma_{j=1,2}\Sigma_{k}X_{ijk}+ \psi\Sigma_{i=3,4,6,8,9}\Sigma_{j=3,4} \Sigma_{k}X_{ijk}}{N}$$

where $\beta_{HCV}^{sex}$ is sexual-related HCV transmission rate and N is the total population size of MSM.

Based on the above two equations, we define the force of infection for HCV as:

$$\Lambda_{k}=\left\{ \begin{aligned} \lambda^{idu}+ \lambda^{sex}, k=2 \\ \lambda^{sex}, k=1,3 \end{aligned} \right.$$

HCV diagnosis rate ($D_{jk}(t)$)

Based on the proportion of MSM non-IDU who were HIV-negative and not on PrEP in 2017 ($\delta_{prop})$, we estimated a rate of HCV diagnosis in this group in 2017 assuming an exponential decay function (details in section 1.2.2). Based on this, we then assume the rate of HCV testing/diagnosis among MSM non-IDU, who were HIV-negative and not on PrEP to start in $D_{start}$, increase linearly until 2017 and then remain constant:

$$\delta(t)=\left\{ \begin{matrix} 0, & t<D_{start} \\ -\frac{\log\left( 1-\delta_{prop} \right)}{2017-D_{start}} (t-D_{start}), & D_{start}\leq t\leq2017 \\ -\log\left( 1-\delta_{prop} \right), & 2017<t \end{matrix} \right.$$

Compared to MSM non-IDU who were HIV-negative and not on PrEP, all other MSM (i.e., ever MSM-IDU or HIV-negative MSM on PrEP or HIV-positive MSM) were assumed to have a higher relative rate of testing ($R_{dHCV}).$We define the rate of HCV diagnosis among all MSM as:

$$D_{jk}(t)=\left\{ \begin{aligned} \delta\left( t \right), j=1, k=1 \\ RR_{dHCV} \delta\left( t \right), j=2,3,4, k=1 \\ {RR}_{dHCV} \delta\left( t \right), j=1\ldots4, k=2,3 \end{aligned} \right.$$

HCV related mortality rate ($M_{j})$

The HCV-related mortality was assumed to be higher among HIV-infected MSM. We define the rate of HCV-related mortality as:

$$M_{j}=\left\{ \begin{aligned} \mu_{HCV}, j=1,2 \\ {RR_{m_{HCV}}\mu}_{HCV}, j=3,4 \end{aligned} \right.$$

HCV treatment rate ($T_{x}\left( t \right)$)

Based on the proportion of HCV-diagnosed MSM treated over a four-year period ${{(T}_{x}}_{Pre-DAA})$, we estimated a yearly rate of HCV treatment in 2012 assuming an exponential decay function (details in section 1.3). Based on this, we then assumed the rate of HCV treatment among diagnosed MSM to start in ${T_{x}}_{start}$, to increase linearly until 2012 to reproduce the 2012 HCV treatment rate, remain stable over 2012-2024, to increase in 2015 by a factor $RR_{Post-DAA},$ and to remain stable afterwards:

$$T_{x}\left( t \right)=\left\{ \begin{matrix} 0, & t<{T_{x}}_{start} \\ \frac{-\frac{\log\left( {{1-T}_{x}}_{Pre-DAA} \right)}{4}}{2012-{T_{x}}_{start}}\left( t-{T_{x}}_{start} \right), & {T_{x}}_{start}\leq t\leq2012 \\ -\frac{\log\left( {{1-T}_{x}}_{Pre-DAA} \right)}{4}, & 2012<t\leq2015 \\ -\frac{\log\left( {{1-T}_{x}}_{Pre-DAA} \right)}{4} RR_{Post-DAA}, & 2015<t \end{matrix} \right.$$

SVR rate (${SVR}_{j}$)

We assume that the proportion of individuals treated for HCV infection who achieve SVR varies as a function of HIV-status until 2015, when DAAs were introduced, and to be the same, regardless of HIV status, from 2015 onward:

$$\tau_{1}, \tau_{2}=\left\{ \begin{matrix} \tau_{1}=\tau_{2}=0, & t<{T_{x}}_{start} \\ \tau_{1}={eff\_preDAAs}_{HIVn}, \tau_{2}={eff\_preDAAs}_{HIVp}, & {T_{x}}_{start}\leq t\leq2015 \\ \tau_{1}=\tau_{2}=eff\_postDAAs, & 2015<t \end{matrix} \right.$$

$${SVR}_{j}=\left\{ \begin{aligned} \tau_{1}, j=1,2 \\ \tau_{2}, j=3,4 \end{aligned} \right.$$

**For movement due to HIV:**

$$B_{i1k} =\left( -S_{1k}-PREP\left( t \right) \right) X_{i1k}+\eta X_{i2k}$$

$$B_{i2k}=\left( {-S}_{2k}-\eta\right) X_{i2k}+PREP(t) X_{i1k}$$

$$B_{i3k}=S_{1k} X_{i1k}-D_{HIV}\left( t \right)X_{i3k}-\mu_{4} X_{i3k}$$

$$B_{i4k}=S_{2k} X_{i2k}+D_{HIV}\left( t \right)X_{i3k}-\left( 1-(t) \right)\mu_{4} X_{i4k}-(t) RR_{ART} \mu_{4} X_{i4k}$$

where:

- $S_{jk}$ is the force of infection for HIV among MSM in HIV infection state *j*, and injecting state *k* (see section below)
- $PREP\left( t \right)$ is the rate of initiation onto PrEP (see section below)
- $\eta$ is the rate of PrEP cessation
- $D_{HIV}\left( t \right)$ is the rate of HIV diagnosis (see section below)
- $(t)$ is the proportion of HIV-positive and diagnosed MSM on ART
- $\mu_{4}$ is the rate of HIV-related mortality among MSM who are not on ART
- $RR_{ART}$ is the relative risk of HIV-related mortality among MSM who are on ART vs MSM who are not on ART

HIV force of infection ($S_{jk}$)

We assume the proportion of HIV-positive and diagnosed MSM who are on ART to start in 1996, increasing linearly until 2017 (at different rates over 1996-2008 and 2008-2017; details in section 1.5), and to remain constant afterwards as:

$$\left( t \right)= \left\{ \begin{matrix} 0, & t<1996 \\ \frac{{}_{1}}{12}(t-1996), & 1996\leq t\leq2008 \\ {}_{1}+\frac{{}_{2}-{}_{1}}{9}\left( t-2008 \right), & 2008<t \leq2017 \\ {}_{2}, & 2017<t \end{matrix} \right.$$

We assume the injecting-related HIV infectivity of MSM on ART is reduced by a factor $.$We define the HIV force of infection due to injecting transmission among recent MSM-IDU not on PrEP as:

$${\sigma_{1}}_{idu}=\beta_{HIV}^{idu} \frac{\sum_{i} X_{i,3,2}+ (t) \Sigma_{i}X_{i,4,2}+\left( 1-(t) \right)\Sigma_{i}X_{i,4,2}}{\Sigma_{ij}X_{i,j,2}}$$

where $\beta_{HIV}^{idu}$ is the injecting-related HIV transmission rate

We assume that the susceptibility to injecting-related HIV acquisition among MSM on PrEP, compared to MSM who are not on PrEP, is reduced by a factor ${}_{1}.$ We define the HIV force of infection due to injecting transmission among recent MSM-IDU on PrEP as:

$${\sigma_{2}}_{idu}= {}_{1}{\sigma_{1}}_{idu}$$

We assume the sexually-related HIV infectivity of MSM on ART is reduced by a factor $.$We define the HIV force of infection due to sexual transmission among all MSM not on PrEP as:

$${\sigma_{1}}_{sex}=\beta_{HIV}^{sex}\frac{\Sigma_{i}\Sigma_{k}X_{i,3,k}+ \left( t \right)\Sigma_{i}\Sigma_{k}X_{i,4,k}+\left( 1-(t) \right)\Sigma_{i}\Sigma_{k}X_{i,4,k})}{N}$$

where $\beta_{HIV}^{sex}$ is the sexually-related HIV transmission rate.

We assume that the susceptibility to sexually-related HIV acquisition among MSM on PrEP, compared to MSM who are not on PrEP, is reduced by a factor ${}_{2}.$ We define the HIV force of infection due to injecting transmission all among MSM on PrEP as:

$${\sigma_{2}}_{sex}= {}_{2}{\sigma_{1}}_{sex}$$

Based on the above equations, we define the force of infection for HIV:

$$S_{jk}=\left\{ \begin{matrix} {\sigma_{1}}_{idu}+{\sigma_{1}}_{sex}, & j=1, k=2 \\ {\sigma_{2}}_{idu}+{\sigma_{2}}_{sex}, & j=2, k=2 \\ {\sigma_{1}}_{sex}, & j=1, k=1,3 \\ {\sigma_{2}}_{sex}, & j=2, k=1,3 \\ 0, & j=3,4 \end{matrix} \right.$$

PrEP initiation rate ($PREP\left( t \right)$)

We assume the rate of PrEP initiation ($PREP\left( t \right))$to increase linearly between 2010-2019 (at different rates in-between the years for which there was data on PrEP coverage) and to remain stable afterwards:

$$PREP\left( t \right)=\left\{ \begin{matrix} 0, & t<2010 \\ \pi_{1}\left( t-2010 \right), & 2010\leq t\leq2011 \\ \pi_{1}+\left( \frac{\pi_{2}}{2} \right)\left( t-2011 \right), & 2011<t\leq2014 \\ \pi_{1}+\pi_{2}+\left( \frac{\pi_{3}}{5} \right)\left( t-2014 \right), & 2014<t\leq2019 \\ \pi_{1}+\pi_{2}+\pi_{3}, & 2019<t \end{matrix} \right.$$

Rate of HIV diagnosis ($D_{HIV}(t)$)

We assume the rate of HIV diagnosis to start in 1985, to increase linearly until 2017, reaching rate $\varepsilon$, and to remain constant afterwards:

$$D_{HIV}(t) \left\{ \begin{matrix} 0, & t<1985 \\ \frac{\varepsilon}{32}\left( t-1985 \right), & 1985\leq t\leq2017 \\ \varepsilon, & 2017<t \end{matrix} \right.$$

**For movement due to injecting drug use, entry into the model and background mortality:**

$$C_{ij1}=-\alpha X_{ij1}-\left( \mu_{1}+\mu_{3} \right)X_{ij1}$$

$$C_{ij2}=\alpha X_{ij1}-(\upsilon+ \mu_{1}+{\mu_{2}+ \mu}_{3}) X_{ij2}$$

$$C_{ij3}=\upsilon X_{ij2}-\left( \mu_{1}+\mu_{3} \right)X_{ij3}$$

$$C_{111}=\mu_{2}\sum_{i, j} X_{ij2}+\left( \mu_{1}+\mu_{3} \right)\sum_{i, j,k} X_{ijk}$$

where:

- $\mu_{1}$is the rate of aging out
- $\mu_{2}$ is the injection drug use-related mortality
- $\mu_{3}$ is the rate of background mortality
- $\alpha$ is the rate at which MSM initiate injection drug use
- $\upsilon$is the rate at which MSM stop injection drug use

# **3.0 CALIBRATION DETAILS**

Calibration was performed using an approximate Bayesian computation sequential Monte Carlo (ABC SMC) scheme, accounting for uncertainty in the calibration data and parameters^[2]^. This scheme starts with 1,000 parameter sets sampled from their prior distributions using Latin Hypercube sampling, which are iteratively perturbed to improve the goodness-of-fit until we achieve our desired fitting criterion, so producing a set of 1,000 baseline model fits. The model fits were used to produce the median and 95% credibility intervals (95% CrI; 2.5th to 97.5th percentile range) for all model projections. Goodness-of-fit was defined as 0 if model projections lie within the calibration range or otherwise, as the sum of the absolute differences on the log scale between the lower or upper value (whichever is closer to the model projections) of the calibration ranges and the corresponding model projections. Our criterion for achieving calibration was defined as a goodness-of-fit score of 0.

# **SUPPLEMENTARY TABLES**

## **Supplementary Table 1:** Data sources

| **Name** | **Eligibility** | **Data** | **Years of available data** | **Recruitment strategy** |
| --- | --- | --- | --- | --- |
| National HIV Behavioural Surveillance survey (NHBS) among MSM | ≥18 years, resided in the San Francisco Metropolitan Statistical Area, and either identified as MSM or had any sex with another man in the previous year | Self-reported behavioural and service use, HIV and HCV antibody testing | 5 rounds, approximately every three years, over 2004-2017; either through published papers (2004 and 2008)^[3, 11, 13]^ or unpublished analyses of data done by our team (2011, 2014 and 2017). | Time-location sampling by intercepting people at venues where gay and other MSM congregate^[14]^ |
| SFAF street-intercept survey | People who self-identify as MSM | Self-reported behavioural and service use | Yearly over 2017-2019 | Convenience: street-based sampling^[15]^ |
| STRUT (electronic health record data) | None. STRUT is frequented by people who self-identify as MSM | Self-reported behavioural and service use | Ongoing over 2017-2019 | Convenience: STRUT is a healthcare centre designed to support the sexual and substance use health of MSM^[15, 16]^ |

## **Supplementary Table 2:** Studies among MSM illustrating the relationships between IDU and sexual risk behaviours with incident or prevalent HCV infection

| **Source** | **Setting/years** | **Findings** |
| --- | --- | --- |
| Clipman et al 2020^[17]^ | Setting: 5 states across India  Years: 2012-2013 | **IDU risk:** When stratified by history of IDU, HCV prevalence was 65% and 0.9% among those with and without this behaviour, respectively.  **Sexual risks:** When stratified by history of unprotected anal intercourse, HIV prevalence was 1.0% and 1.5%, among those with and without this behaviour, respectively. |
| Hoornenborg et al 2020^[18]^ | Setting: Amsterdam  Years: 2015-2018 | **IDU risk:** MSM who reported past-year IDU had 4.7 (95%CI: 1.6-12.1) greater risk of acquiring HCV compared to those who did not report past-year IDU.  **Sexual risks:** Several sexual risk factors were explored in relation to HCV acquisition, including number of receptive condomless anal sex acts, having had anal sex with ≥1 HIV-positive partner, chemsex and sharing of sex toys. In multivariable analyses, the relative risks for these behaviours ranged from 1.5 (95%CI: 0.6-3.9) for fisting without gloves to 2.0 (95%CI: 0.8-4.9) for chemsex. |
| Mata-Marin et al 2022^[19]^ | Setting: Mexico City, Mexico  Years: 2019 | **IDU risk**: MSM who reported IDU had 2.6 (95%CI: 1.1-6.6) greater odds of testing HCV antibody positive compared to those not reporting IDU.  **Sexual risks:** Several sexual risk factors were explored in relation to testing HCV antibody positive, including chemsex, sharing sex toys, group sex and enemas previous to sex. The odds ratios for these behaviours ranged from 1.1 (95%CI: 0.4-2.4) for receptive fisting to 6.2 (95% CI: 2.3-16.4) for sharing sex toys. MSM who reported chemsex had 1.5 (95% CI: 0.9-2.6) greater odds of testing HCV antibody positive compared to those not reporting chemsex. |
| Vanhommerig et al 2015^[20]^ | Setting: Amsterdam, The Netherlans  Years: 2009-2014 | **IDU risk**: MSM who reported IDU had 42.9 (95%CI: 5.0-368.8) greater odds of being diagnosed with acute HCV than those who did not report IDU.  **Sexual risks:** Several sexual risk factors were explored in relation to being diagnosed with acute HCV, including insertive/receptive unprotected anal intercourse, sharing sex toys and group sex. The odds ratios for these behaviours ranged from 1.2 (95%CI: 0.3-5.0) for only engaging in insertive unprotected anal intercourse to 12.4 (95% CI: 4.5-33.8) for sharing sex toys. |
| Witt et al 2013^[21]^ | Setting: 4 metropolitan areas in the US  Years: 1980s-2011 | **IDU risk:** In multivariable analyses, compared to MSM who reported never IDU and no recreational drug use, those who only reported recreational drug use had a 1.4 (95%CI: 0.9-2.1) higher risk of HCV acquisition. Compared to the same reference group, those who reported ever IDU had a 4.7 (95% CI: 2.1-10.5) higher risk of HCV acquisition.  **Sexual risks:** Several sexual risk factors were explored in relation to HCV acquisition, including having multiple sexual partners and unprotected receptive anal intercourse. In multivariable analyses, the relative risks for these behaviours ranged from 1.4 (95%CI: 0.8-2.5) for engaging in unprotected receptive anal intercourse with ≤1 partner to 3.4 (95% CI: 1.7-6.7) for engaging in unprotected receptive anal intercourse with >1 partner. |

## **Supplementary Table 3:** Prior ranges for parameters used in the model

| **PARAMETER** | **PRIORS: DISTRIBUTION AND ESTIMATES** | **SOURCES AND COMMENTS** |
| --- | --- | --- |
| **Demographic characteristics** | | |
| MSM population size in 1985 | Uniform:  65,523 – 140,000 | The lower bound reflects the lower bound of the 2017 estimate of the MSM population size in San Francisco^[22]^. We set the upper bound higher, as the population is expected to have decreased since 1985 due to HIV-related mortality in a context with high HIV prevalence and no treatment until 1996^[12]^. It was set to 120,000 initially and since the posterior values selected by the model were close to this threshold, it was subsequently increased to 140,000. |
| Injection drug use-related mortality (per person per year) | Uniform:  0.00088 - 0.0364 | In the absence of data on non-HIV related mortality among recent MSM-IDU, we triangulated different data sources to estimate it: (i) in a systematic review, mortality among HIV-negative people who inject drugs in North America (n= 3 studies for this sub-group) was estimated at 1.73 per 100py (95% CI: 0.176 – 3.64)^[23]^; (ii) in a separate systematic review, overdose mortality rate in people who inject primarily stimulants compared to those injecting primarily opioids was 54% lower (RR: 0.54, 95%CI: 0.22 – 1.33)^[24]^; (iii) in the 2017 NHBS survey, 64.5% of MSM-IDU reported meth/amphetamine as the main drug injected^[25]^. We therefore reduced the lower bound of the estimate of the estimate in (i) by 50%. |
| Background mortality (per person per year) | Point estimate:  0.0087 | All-cause mortality rate in 2019 for San Francisco in the general population^[26]^. |
| HIV-related mortality if MSM are not on ART (per person per year) | Uniform:  0.082-0.096 | Based on a pooled analysis from Europe, North America and Australia, the median duration of survival among HIV-infected MSM who are not treated is 11.2 (10.4-12.2) years^[27]^. |
| Relative risk of HIV-related mortality among MSM who are on ART vs MSM who are not on ART | Uniform:  0.232 – 0.348 | If infected with HIV, the expected survival of 35-year old men on ART treatment is 73^[28]^, so 38 years compared to 11.2^[27]^ years among those who are not on ART. We therefore assumed the relative risk of HIV-related death among those on ART compared to those who are not on ART is 11.2/38 = 0.29. We then added 20% uncertainty around this estimate. |
| HCV-related mortality among MSM who are not infected with HIV (per person per year) | Triangular:  0.0053  (0.0013 – 0.0093) | Based on a prospective cohort study using NHANES data in the United States which found the difference in the rates of liver related mortality between HCV Ab+/RNA+ and HCV Ab-^[29]^. |
| Relative risk of HCV-related mortality among MSM who are co-infected with HIV | Lognormal:  2.1 (1.5 – 3.0) | Based on the rate ratio of cirrhosis development in HIV/HCV co-infected vs HCV mono-infected individuals^[30]^. We assumed the relative risk increase to be the same among individuals on ART and those who are not, given little evidence to suggest otherwise^[30]^. |
| **HCV-related parameters** | | |
| HCV seed (i.e., proportion of participants chronically infected with HCV) among ever MSM-IDU (recent and non-recent) in 1985 | Uniform:  0.061 – 0.256 | Informed by an estimate of HCV Ab prevalence in 1985-1986 among MSM with a history of injection drug use enrolled in the San Francisco General Hospital cohort study and the San Francisco Men’s Health Study: 18.1% (95% CI: 12.1% - 25.6%)^[31]^. We reduced the lower bound by 50% because the study over-sampled HIV-positive MSM, who have a higher prevalence of HCV relative to HIV-negative MSM^[4]^. |
| HCV seed among MSM non-IDU in 1985 | Uniform:  0.007 - 0.029 | Informed by an estimate of HCV Ab prevalence in 1985-1986 among MSM with no history of injection drug use enrolled in the San Francisco General Hospital cohort study and the San Francisco Men’s Health Study: 1.5% (95% CI: 0.7% - 2.9%)^[31]^. |
| Sexually-related HCV transmission rate (per person per year) | Uniform:  0 – 0.5 | Uninformative prior; varied to calibrate to HCV prevalence |
| Relative risk of HCV transmission through injection drug use practices vs sexual practices | Uniform:  2 - 10 | Wide range informed by studies estimating the magnitude of the associations between IDU and sexual risk behaviours and incident or prevalent HCV infection (Supplementary material-Table S2); varied to calibrate to HCV prevalence |
| Relative risk of HCV infectivity among MSM who are HIV-infected vs MSM who are not HIV-infected | Lognormal:  2.6 (95% CI: 1.50 – 4.43) | This estimate was used in previous modelling studies among MSM^[32, 33]^ and other populations^[34]^. In the absence of MSM-specific data, it is based on a systematic review and meta-analysis comparing the risk of mother-to-child HCV transmission in HIV+ and HIV-mothers^[35]^.This finding is corroborated by two other studies: one which found HIV co-infection to increase the needlestick HCV transmission probability (2.8 fold, 95% CI, 0.5-10.8)^[36]^ and another which found the needlestick HCV transmission probability was increased at higher HCV viral loads (11 fold (95% CI 1.1-114.1) for ~4 log increase in HCV viral load)^[37]^ which are known to increase if an individual is HIV co-infected^[38-42]^. |
| Proportion of individuals who spontaneously clear HCV infection among HIV negative MSM | Uniform:  0.22 - 0.29 | ^[43]^ |
| Proportion of individuals who spontaneously clear HCV infection among HIV positive MSM | Uniform:  0.12 - 0.19 | ^[44]^ |
| Year HCV testing started | Uniform:  1999-2001 | Assumption based on the high level of HCV testing reported by MSM enrolled in NHBS 2004 and 2008^[3]^, which reflects the earliest years for which data are available. Details are provided in Supplementary material (section 1.2). |
| Proportion of MSM non-IDU, who were HIV-negative and not on PrEP and reported HCV testing in the past year | Normal:  17.3%  (12.2% - 23.4%) | Based on NHBS 2017 (unpublished). Used to inform the rate of HCV testing. Details are provided in Supplementary material (section 1.2). |
| Relative risk of HCV testing among MSM who belonged to any of the following groups, compared to MSM non-IDU who were HIV-negative and not on PrEP   - ever MSM-IDU or - HIV negative MSM on PrEP or - HIV positive MSM | Lognormal:  2.5 (1.8 – 3.5) | Based on differences in HCV testing by injection and HIV infection statues among MSM in NHBS 2017 (unpublished). Details are provided in Supplementary material (section 1.2). |
| Year interferon-based HCV treatment started | Uniform:  2002 - 2004 | ^[8]^ |
| Year DAAs were introduced | Point estimate:  2015 | ^[45]^ |
| Proportion of HIV-positive MSM who reported ever treatment over 2008-2014 | Lognormal  15.7% (12.8% - 18.9%) | ^[5]^ Used to inform the rate of HCV treatment before the introduction of DAAs. |
| Relative risk reflecting the increase in rate of HCV treatment in 2015 due to DAA scale-up relative to previous years | Uniform  1-10 |  |
| Rate of exit from the HCV treatment compartment during the interferon era (2004-2014; per person per year) | Point estimate:  52/48 | Taken as the inverse of the average duration of treatment with interferon: 48 weeks^[46]^. |
| Rate of exit from the HCV treatment compartment during the DAA era (2015-onward; per person per year) | Uniform:  52/8 —52/12 | Taken as the inverse of the average duration of treatment with DAAs: 8-12 weeks^[47]^. |
| Proportion of HCV treatments that result in SVR during the interferon era (2004-2014) among HIV-negative MSM | Normal:  64% (59%- 69%) | Observational study examining the efficacy of interferon- and ribavirin-based HCV treatment in HIV-negative participants^[48]^. |
| Proportion of HCV treatments that result in SVR during the PEG-IFN era (2004-2014) among HIV-positive MSM | Normal:  38% (35%-42%) | Meta-analysis of observational studies examining the efficacy of interferon- and ribavirin-based HCV treatment in HIV-positive participants^[46]^. |
| Proportion of HCV treatments that result in SVR during the DAA era (2015-onward) irrespective of HIV status | Uniform:  90% - 100% | Based on a review of observational studies on the efficacy of DAAs^[49]^. |
| **HIV-related parameters** | | |
| HIV seed in 1985 among all MSM | Uniform:  0.25 – 0.5 | Informed by an estimate of HIV prevalence among MSM enrolled in the San Francisco Men’s Health Study in 1985: 49.5% (95% CI: 46.7% - 52.3%)^[50]^. We reduced the lower bound to 25% because the study recruited participants from areas in San Francisco most affected by the HIV epidemic, and this could have over-estimated the overall HIV prevalence. |
| Injection drug use-related HIV transmission rate (per person per year) | Uniform:  0 – 0.5 | Uninformative prior; varied to calibrate to HIV prevalence |
| Sexually-related HIV transmission rate (per person per year) | Uniform:  0 – 0.5 | Uninformative prior; varied to calibrate to HIV prevalence |
| Year HIV testing started | Point estimate:  1985 | ^[12]^ |
| Rate at which individuals are tested for HIV (per person per year) | Uniform:  0-1 | Varied to calibrate to proportion of HIV-positive MSM who are diagnosed over time (Table 2). We assume that the rate of HIV testing increased linearly between 1985 and 2017 (the most recent year for which data are available). We also assume that the HIV testing rate does not vary by injection drug use status but that MSM on PrEP are diagnosed right away given results based on data from NHBS 2017. Details are provided in Supplementary material (section 1.4). |
| Year ART started | Point estimate:  1996 | ^[12]^ |
| Proportion of HIV positive and diagnosed MSM who are on ART | Normal distribution  2008: 79.3%  (70.6% - 87.3%)  2017: 93.8%  (86.7% – 97.8%) | Based on NHBS 2008^[11]^ and NHBS and SFAF MSM street-intercept study 2017 (unpublished data); used to inform the rate of HIV treatment, which was assumed to have scaled-up linearly between 1996 and 2017 and to have remained stable afterwards; rate assumed to be the same regardless of injecting status. Details are provided in Supplementary material (section 1.5). |
| Relative risk reflecting reduced HIV infectivity among MSM on ART vs those who are not on ART | Lognormal:  0 (95% CI: 0.0-0.23) | Based on a prospective observational study done at 75 sites in 14 European countries which explored sexual HIV transmission among serodiscordant MSM partners with the HIV-positive partner taking ART^[51]^. Results are similar to those of a multi-country (including the United States) RCT, which compared risk of linked sexual partner HIV transmission in participants on early ART vs delayed ART^[52]^. |
| Relative risk reflecting the reduced susceptibility to injection drug use-related HIV acquisition among MSM on PrEP vs those not on PrEP | Lognormal:  0.51 (0.28 – 0.90) | Based on the only RCT (Bangkok trial) to have examined the relative risk of HIV transmission as a function of PrEP receipt among people who inject drugs^[53]^. |
| Relative risk reflecting the reduced susceptibility to sexually-related HIV acquisition among MSM on PrEP vs those not on PrEP | Lognormal  0.23 (0.08 – 0.62) | Based on a systematic review and meta-analysis of RCT (n= 4 studies) examining the effect of PrEP among MSM^[54]^. |
| Year PrEP started | Point estimate:  2010 | Truvada® was approved by the Food and Drug Administration (FDA) in 2012.^[55]^ Based on published data from NHBS, PrEP use was 0% in 2004 and 2008, and 1.4% in 2011^[13]^; before the FDA approval, a small number of people were able to access PrEP through clinical trials. |
| Rate at which HIV-negative susceptible individuals start using PrEP (per person per year) | Uniform:  0-3 | Varied to calibrate to proportion of HIV-negative MSM who are on PrEP over time using NHBS and SFAF MSM street-intercept study data (Table 2). Assumed to increase linearly between 2010-2019 (at different rates in-between the years for which there was data on PrEP coverage through the NHBS surveys) and remain stable afterwards. We assumed the same rate of PrEP initiation regardless of injecting status based on NHBS 2017 data. Details are provided in Supplementary material (section 1.6). |
| Rate at which HIV-negative susceptible individuals stop using PrEP (per person per year) | Triangular:  1.47 (1.22 – 1.82) | Data from San Francisco Primary Care Clinics, a 15-clinic municipal health network found median continued use of PrEP to be 8.2 months over 2012-2017 (a majority of participants were MSM). No range was given so we assume a +/- 20% variation on this of 6.6-9.8 months^[56]^. |
| **Injection drug use related parameters** |  |  |
| The rate at which MSM initiate injection drug use (per person per year) | Uniform:  0 - 0.3 | Uninformative prior; varied to calibrate to the proportion of MSM who are recent, non-recent and MSM non-IDU over time in NHBS (Table 2). |
| Rate at which MSM stop injection drug use (per person per year) | Uniform:  0.01 - 0.5 | Uninformative prior; varied to calibrate to the proportion of MSM who are recent MSM-IDU, non-recent MSM-IDU and MSM non-IDU over time in NHBS (Table 2). |

## **Supplementary Table 4:** Data used to calibrate and validate the model

| **Name** | **Estimate**  **(95% CI)** | **Date of**  **estimate** | **Data source** |
| --- | --- | --- | --- |
| **CALIBRATION DATA** | | | |
| MSM population size | 69,974  (65,523–74,323) | 2017 | Pooled estimate informed by several studies^[57]^ |
| HCV Ab prevalence among ever MSM-IDU | 15.5% (7.3% - 23.9%) | 2011 | Published NHBS-MSM estimate^[4]^ |
| HCV Ab prevalence among MSM non-IDU | 2.3% (0.8% - 3.8%) | 2011 | Published NHBS-MSM estimate^[4]^ |
| HCV Ab prevalence among HIV+ MSM | 15.2% (7.7%-22.7%) | 2004 | Published NHBS-MSM estimate^[3]^ |
|  | 8.3% (3.3% - 13.2%) | 2008 | Published NHBS-MSM estimate ^[3]^ |
|  | 15.7% (8.8-22.7%) | 2011 | Published NHBS-MSM estimate^[4]^ |
| HCV Ab prevalence among HIV- ever MSM-IDU | 9.5% (2.7% - 22.6%) | 2011 | Published NHBS-MSM estimate^[4]^ |
| HIV prevalence among ever MSM-IDU | 47.3% (35.6% - 59.3%) | 2011 | Unpublished NHBS-MSM estimate |
|  | 44.1% (27.2% - 62.1%) | 2014 | Unpublished NHBS-MSM estimate |
|  | 32.8% (21.3% - 46.0%) | 2017 | Unpublished NHBS-MSM estimate |
| HIV prevalence among MSM non-IDU | 18.8% (15.1% - 23.0%) | 2011 | Unpublished NHBS-MSM estimate |
|  | 17.4% (13.4% - 22.0%) | 2014 | Unpublished NHBS-MSM estimate |
|  | 17.3% (13.8% - 21.3%) | 2017 | Unpublished NHBS-MSM estimate |
| Proportion of HCV diagnosed MSM who were ever treated | 63.6% (45.1% - 79.6%) | 2018 | Pooled estimates derived from NHBS-MSM and SFAF-S. Details are provided in Supplementary material (section 1, pp5) |
| Proportion of HIV-positive MSM who are diagnosed | 78.3% (69.8% - 86.8%) | 2004 | Published NHBS-MSM estimate^[11]^ |
|  | 82.0% (74.8 - 89.1%) | 2008 | Published NHBS-MSM estimate^[11]^ |
|  | 92.7% (86.1% - 96.8%) | 2011 | Unpublished NHBS-MSM estimate |
|  | 95.7% (88.9% - 99.1%) | 2014 | Unpublished NHBS-MSM estimate |
|  | 95.7% (89.4% -98.8%) | 2017 | Unpublished NHBS-MSM estimate |
| Proportion of HIV negative MSM on PrEP | 1.4% (0.4% - 3.2%) | 2011 | Published NHBS-MSM estimate^[13]^ |
|  | 9.8% (6.6% - 13.7%) | 2014 | Published NHBS-MSM estimate^[13]^ |
|  | 41.8% (37.1% - 46.7%) | 2017 | Pooled estimate derived from NHBS-MSM 2017 and SFAF MSM street-intercept study 2017. NHBS-MSM: 44.9% (39.9% - 50.0%)^[13]^  SFAF MSM street-intercept study (unpublished): 39.2% (34.7% – 43.9%). |
|  | 39.4% (29.4% - 50.0%) | 2018 | Unpublished SFAF MSM street-intercept study estimate |
|  | 45.3% (36.5% - 54.4%) | 2019 | Unpublished SFAF MSM street-intercept study estimate |
| Proportion of MSM who injected in the previous year (recent MSM-IDU) | 4.3% (2.4% - 7.0%) | 2014 | Unpublished NHBS-MSM estimate |
|  | 6.0% (4.1% - 8.5%) | 2017 | Unpublished NHBS-MSM estimate |
| Proportion of MSM who ever injected but not in the previous year (non-recent MSM-IDU) | 5.4% (3.3% - 8.4%) | 2014 | Unpublished NHBS-MSM estimate |
|  | 6.7% (4.6% - 9.2%) | 2017 | Unpublished NHBS-MSM estimate |
| Proportion of MSM who never injected (MSM non-IDU) | 90.3 % (86.7% - 93.2%) | 2014 | Unpublished NHBS-MSM estimate |
|  | 87.3% (84.0% - 90.2%) | 2017 | Unpublished NHBS-MSM estimate |
| **VALIDATION DATA** | | | |
| HCV Ab prevalence among all MSM | 4.5% (2.6% - 6.4%) | 2011 | Published NHBS estimate^[4]^ |
| HCV Ab prevalence among HIV+ ever MSM-IDU | 34.8% (18.7% - 55.2%) | 2004 | Published NHBS estimate^[3]^ |
|  | 17.6% (13.9 – 31.9) | 2008 | Published NHBS estimate^[3]^ |
|  | 22.9% (8.2% - 37.5%) | 2011 | Published NHBS estimate^[4]^ |
| HCV Ab prevalence among HIV+ MSM non-IDU | 8.7% (1.9% - 15.5%) | 2004 | Published NHBS estimate^[3]^ |
|  | 4.5% (0.1% - 8.9%) | 2008 | Published NHBS estimate^[3]^ |
|  | 12.3% (4.6% - 20.1%) | 2011 | Published NHBS estimate^[4]^ |
| HCV Ab prevalence among HIV- MSM non-IDU | 1.6% (0.8% - 2.8%) | 1997-2000 | Published estimate among MSM seeking repeat anonymous HIV testing in San Francisco^[58]^ |
| HIV prevalence, all MSM | 24.0% (19.6% - 28.1%) | 2004 | Published NHBS estimate^[11]^ |
|  | 23.0% (19.0% - 26.3%) | 2008 | Published NHBS estimate^[11]^ |
|  | 23.0% (18.9% - 26.6%) | 2011 | Published NHBS estimate^[11]^ |
|  | 20.0% (15.9% - 24.6%) | 2014 | Unpublished NHBS estimate |
|  | 19.3 (15.9% - 23.1%) | 2017 | Unpublished NHBS estimate |
| Proportion of MSM ever chronically infected with HCV who were ever diagnosed | 66.7% (43.0% - 85.4%) | 2011 | Published NHBS estimate^[4]^ |
| HIV incidence among all MSM | 4.7 per 100py  (2.8 – 7.3) | 1985 | Published estimates among MSM followed in the San Francisco Men’s Health Study^[59]^ |
|  | 4.2 per 100py  (2.4 – 6.9) | 1986 | Published estimates among MSM followed in the San Francisco Men’s Health Study^[59]^ |
|  | 1.2 per 100py  (0.4 – 2.9) | 1987 | Published estimates among MSM followed in the San Francisco Men’s Health Study^[59]^ |
|  | 2.2 per 100py  (1.1 – 4.0) | 1996 | Published estimates among MSM seeking anonymous testing at AIDS Health Project, the largest provider of HIV testing in the city^[60]^ |
|  | 1.3 per 100py  (0.5 – 2.7) | 1997 | seeking anonymous testing at AIDS Health Project, the largest provider of HIV testing in the city^[60]^ |
|  | 2.1 per 100py  (1.0 – 4.1) | 1998 | seeking anonymous testing at AIDS Health Project, the largest provider of HIV testing in the city^[60]^ |
|  | 4.2 per 100py  (2.2 – 7.6) | 1999 | seeking anonymous testing at AIDS Health Project, the largest provider of HIV testing in the city^[60]^ |
|  | 2.6 per 100py  (0.8 - 4.3) | 2004 | Published NHBS estimates^[11]^ |
|  | 0.7 per 100py  (0 - 1.5) | 2008 | Published NHBS estimates^[11]^ |
|  | 1.0 per 100py  (0.02 – 1.9) | 2011 | Published NHBS estimates^[11]^ |
| Proportion of MSM who injected in the previous year (recent MSM-IDU) | 4.3% (3.8% - 4.8%) | 2017 | Unpublished STRUT estimate |
|  | 7.2% (6.3% - 8.1%) | 2018 | Unpublished STRUT estimate |
|  | 5.7% (4.6% - 6.9%) | 2019 | Unpublished STRUT estimate |

## **Supplementary Table 5:** Changes in HIV and HCV services due to the COVID-19 pandemic: data and assumptions

| **Model parameter(s)** | **Assumed change due to the COVID-19 pandemic** | **Source(s)** |
| --- | --- | --- |
| Rate of HCV testing and rate of HCV treatment uptake among chronic diagnosed MSM | For each rate, we assumed a decrease of 59% over March – December 2020 compared to pre-March 2020 levels | In a recent study, the average number of monthly HCV Ab tests provided by different community partners in San Francisco was found to have decreased from 702.3 over March 2019 – February 2020 to 287.3 over March 2020 – December 2020, suggesting a 59% decrease due to the COVID-19 pandemic^[61]^. In a US-wide study using data from two large national labs (Quest and LabCorp), HCV Ab testing, HCV RNA testing and HCV treatment uptake were each found to have decreased by approximately the same extent (31%-38%) over March – April 2020 compared to the pre-COVID period^[62]^. By December 2020, HCV Ab testing levels had recovered to pre-pandemic levels, whereas HCV RNA testing and HCV treatment uptake had not^[62]^. |
| Rate of HIV testing and proportion of HIV-diagnosed MSM receiving ART | For each parameter, we assumed a decrease of 31% over March – June 2020 compared to pre-March 2020 levels | In San Francisco, the number of laboratory-based HIV testing has decreased at different levels across different organisations over January – June 2020 (median: 33%; range: 2% - 64%)^[63]^. |
| Rate of PrEP initiation | We assumed the rate to have decreased by 34.9% over March 2020 – March 2021 compared to pre-March 2020 levels | In a US-wide study using data from the IQVIA Real World Data—Longitudinal Prescriptions Database, the number of new PrEP users in the state of California decreased by 34.9% over March 2020 – March 2021 compared to pre-COVID levels^[64]^. This data is a national pharmacy database that captures prescriptions from all payers and represents approximately 92% of all prescriptions dispensed from retail pharmacies and 60-86% from mail order outlets in the US^[64]^. |

## **Supplementary Table 6:** Results**—**Modelled incidence and chronic prevalence in 2015

| **Outcome*/group** | **Median (95% CrI)** |
| --- | --- |
| **HCV incidence** |  |
| All | 0.28 (0.23 - 0.35) |
| Recent MSM-IDU | 2.52 (1.69 - 3.60) |
| Non-recent MSM-IDU | 0.17 (0.14 - 0.20) |
| MSM non-IDU | 0.17 (0.14 - 0.20) |
| Ever MSM-IDU | 1.18 (0.84 - 1.57) |
| **Primary HCV incidence** |  |
| All† | 0.28 (0.23 – 0.34) |
| **HCV reinfection** |  |
| All† | 0.64 (0.43 – 0.91) |
| **Chronic HCV prevalence** |  |
| All | 4.45 (4.13 - 4.78) |
| Recent MSM-IDU | 16.71 (13.25 - 19.97) |
| Non-recent MSM-IDU | 16.73 (14.68 - 18.62) |
| MSM non-IDU | 2.66 (2.33 - 2.86) |
| Ever MSM-IDU | 16.77 (14.84 - 18.19) |

*Units for incidence and prevalence are /100 person-years and %, respectively.

†Primary and HCV reinfection data are only presented among all MSM because rates are assumed to be the same when stratified by IDU status. Of all MSM at risk of HCV acquisition, 98.5% were at risk of primary HCV and 1.5% were at risk of HCV reinfection.

Abbreviation: CrI= credible interval.

## **Supplementary Table 7:** Results**—**Modelled incidence and chronic prevalence in 2022 and relative reduction over 2015-2022, depending on the level of recovery in COVID-19 related service disruptions

|  | **Status quo.**  No recovery in COVID-19 related disruptions | **Scenario 1: Slow recovery.**  Recovery in COVID-19 related disruptions by 2025 | **Scenario 2: Rapid recovery.**  Recovery in COVID-19 related disruptions by 2022 |
| --- | --- | --- | --- |
| **Outcome*/group** | **Median (95% CrI)** | **Median (95% CrI)** | **Median (95% CrI)** |
| **HCV incidence, 2022 (per 100 person-years)** | | | |
| All | 0.08 (0.05 - 0.14) | 0.08 (0.05 - 0.14) | 0.08 (0.05 - 0.13) |
| Recent MSM-IDU | 0.71 (0.40 - 1.32) | 0.70 (0.39 - 1.31) | 0.68 (0.38 - 1.27) |
| Non-recent MSM-IDU | 0.05 (0.03 - 0.08) | 0.05 (0.03 - 0.07) | 0.04 (0.03 - 0.07) |
| MSM non-IDU | 0.05 (0.03 - 0.08) | 0.05 (0.03 - 0.07) | 0.04 (0.03 - 0.07) |
| Ever MSM-IDU | 0.33 (0.19 - 0.60) | 0.33 (0.18 - 0.59) | 0.31 (0.17 - 0.57) |
| **Chronic HCV prevalence, 2022 (%)** | | | |
| All | 1.34 (0.96 - 1.99) | 1.32 (0.94 - 1.97) | 1.26 (0.89 - 1.91) |
| Recent MSM-IDU | 5.12 (3.31 - 8.25) | 5.05 (3.26 - 8.18) | 4.84 (3.12 - 7.94) |
| Non-recent MSM-IDU | 3.82 (2.55 - 6.18) | 3.75 (2.49 - 6.10) | 3.53 (2.33 - 5.89) |
| MSM non-IDU | 0.88 (0.64 - 1.26) | 0.86 (0.63 - 1.25) | 0.83 (0.60 - 1.21) |
| Ever MSM-IDU | 4.38 (2.91 - 7.01) | 4.31 (2.85 - 6.93) | 4.10 (2.69 - 6.67) |
| **Relative reduction in HCV incidence, 2022-2015 (%)** | | | |
| All | 71.12 (56.48 - 79.03) | 71.50 (56.87 - 79.37) | 72.67 (58.02 - 80.35) |
| Recent MSM-IDU | 71.99 (57.78 - 79.24) | 72.34 (58.16 - 79.56) | 73.43 (59.30 - 80.51) |
| Non-recent MSM-IDU | 72.90 58.82 - 80.65) | 73.28 (59.22 - 80.98) | 74.39 (60.43 - 81.94) |
| MSM non-IDU | 72.90 (58.82 - 80.65) | 73.28 (59.22 - 80.98) | 74.39 (60.43 - 81.94) |
| Ever MSM-IDU | 72.34 (57.98 - 79.65) | 72.71 (58.37 - 79.97) | 73.79 (59.52 - 80.90) |
| **Relative reduction in chronic HCV prevalence, 2022-2015 (%)** | | | |
| All | 69.97 (55.68 - 78.06) | 70.41 (56.15 - 78.46) | 71.72 (57.56 - 79.62) |
| Recent MSM-IDU | 69.30 (54.90 - 77.06) | 69.72 (55.35 - 77.45) | 70.96 (56.70 - 78.59) |
| Non-recent MSM-IDU | 76.99 (62.79 - 84.47) | 77.41 (63.24 - 84.83) | 78.64 (64.61 - 85.86) |
| MSM non-IDU | 66.87 (53.14 - 75.01) | 67.34 (53.62 - 75.43) | 68.70 (55.02 - 76.66) |
| Ever MSM-IDU | 73.64 (59.48 - 81.51) | 74.06 (59.94 - 81.88) | 75.33 (61.33 - 82.95) |

*Units for incidence and prevalence are /100 person-years and %, respectively.

Abbreviation: CrI= credible interval

## **Supplementary Table 8:** Results**—**Projected HCV incidence and chronic HCV prevalence in 2030 and relative change over 2015-2030

|  | **Status quo.**  No recovery in COVID-19 related disruptions | **Scenario 1:**  **Slow recovery.**  Recovery in COVID-19 related disruptions by 2025 | **Scenario 2:**  **Rapid recovery.**  Recovery in COVID-19 related disruptions by 2022 | **Scenario 3: Rapid plus.**  Scenario 2 + increase HCNSP to 100% | **Counterfactual:** Remove HCV testing and treatment over 2023-2030. | **Counterfactual:**  No COVID-19 |
| --- | --- | --- | --- | --- | --- | --- |
| **Outcome*/group** | **Median (95% CrI)** | **Median (95% CrI)** | **Median (95% CrI)** | **Median (95% CrI)** | **Median (95% CrI)** | **Median (95% CrI)** |
| **HCV incidence, 2030 (per 100 person-years)** | | | | | | |
| All | 0.04 (0.02 - 0.09) | 0.02 (0.01 - 0.05) | 0.01 (0.01 - 0.04) | 0.01 (0.01 - 0.03) | 0.07 (0.04 - 0.13) | 0.01 (0.01 - 0.04) |
| Recent MSM-IDU | 0.39 (0.19 - 0.88) | 0.16 (0.07 - 0.46) | 0.13 (0.05 - 0.39) | 0.08 (0.04 - 0.26) | 0.62 (0.33 - 1.29) | 0.11 (0.04 - 0.35) |
| Non-recent MSM-IDU | 0.02 (0.01 - 0.05) | 0.01 (0.00 - 0.02) | 0.01 (0.00 - 0.02) | 0.01 (0.00 - 0.02) | 0.04 (0.03 - 0.07) | 0.01 (0.00 - 0.02) |
| MSM non-IDU | 0.02 (0.01 - 0.05) | 0.01 (0.00 - 0.02) | 0.01 (0.00 - 0.02) | 0.01 (0.00 - 0.02) | 0.04 (0.03 - 0.07) | 0.01 (0.00 - 0.02) |
| Ever MSM-IDU | 0.18 (0.08 - 0.40) | 0.07 (0.03 - 0.21) | 0.06 (0.02 - 0.18) | 0.04 (0.02 - 0.12) | 0.29 (0.15 - 0.58) | 0.05 (0.02 - 0.16) |
| **Chronic HCV prevalence (%)** | | | | | | |
| All | 0.74 (0.45 - 1.34) | 0.30 (0.15 - 0.71) | 0.24 (0.12 - 0.61) | 0.23 (0.11 - 0.58) | 1.26 (0.86 - 1.97) | 0.20 (0.10 - 0.53) |
| Recent MSM-IDU | 3.01 (1.68 - 5.92) | 1.24 (0.57 - 3.08) | 1.00 (0.45 - 2.66) | 0.86 (0.39 - 2.27) | 4.86 (2.91 - 8.24) | 0.83 (0.36 - 2.35) |
| Non-recent MSM-IDU | 2.01 (1.15 - 4.01) | 0.72 (0.34 - 1.98) | 0.57 (0.25 - 1.70) | 0.56 (0.25 - 1.67) | 3.85 (2.52 - 6.33) | 0.47 (0.20 - 1.49) |
| MSM non-IDU | 0.48 (0.30 - 0.84) | 0.20 (0.11 - 0.44) | 0.16 (0.08 - 0.38) | 0.16 (0.08 - 0.37) | 0.80 (0.56 - 1.21) | 0.13 (0.07 - 0.33) |
| Ever MSM-IDU | 2.44 (1.36 - 4.80) | 0.94 (0.43 - 2.42) | 0.76 (0.33 - 2.09) | 0.69 (0.30 - 1.89) | 4.30 (2.69 - 7.17) | 0.62 (0.27 - 1.82) |
| **Relative reduction in HCV incidence, 2030-2015 (%)** | | | | | | |
| All | 84.41 (71.34 -90.42) | 93.63 (84.36 - 96.69) | 94.84 (86.46 - 97.44) | 95.82 (89.17 - 97.91) | 74.03 (58.93 - 82.27) | 95.72 (88.13 - 97.95) |
| Recent MSM-IDU | 84.70 (72.03 - 90.48) | 93.70 (84.69 - 96.64) | 94.89 (86.69 - 97.40) | 96.59 (91.33 - 98.28) | 75.22 (60.73 - 82.89) | 95.74 (88.25 - 97.92) |
| Non-recent MSM-IDU | 86.02 (73.69 - 91.33) | 94.35 (86.16 - 97.08) | 95.45 (88.11 - 97.75) | 95.64 (88.66 - 97.85) | 75.94 (61.47 - 83.39) | 96.24 (89.62 - 98.21) |
| MSM non-IDU | 86.02 (73.69 - 91.33) | 94.35 (86.16 - 97.08) | 95.45 (88.11 - 97.75) | 95.64 (88.66 - 97.85) | 75.94 (61.47 - 83.39) | 96.24 (89.62 - 98.21) |
| Ever MSM-IDU | 84.89 (72.25 - 90.75) | 93.85 (84.91 - 96.77) | 95.01 (86.90 - 97.51) | 96.57 (91.17 - 98.29) | 75.47 (61.04 - 83.19) | 95.86 (88.45 - 98.01) |
| **Relative reduction in chronic HCV prevalence, 2030-2015 (%)** | | | | | | |
| All | 83.41 (70.33 - 89.45) | 93.29 (84.40 - 96.41) | 94.57 (86.59 - 97.23) | 94.81 (87.14 - 97.36) | 71.69 (56.67 - 80.08) | 95.50 (88.22 - 97.80) |
| Recent MSM-IDU | 82.09 (68.22 - 88.61) | 92.58 (82.55 - 95.98) | 93.95 (84.91 - 96.86) | 94.79 (87.24 - 97.34) | 71.11 (55.62 - 79.61) | 94.95 (86.74 - 97.49) |
| Non-recent MSM-IDU | 87.86 (76.07 - 93.05) | 95.63 (88.32 - 98.00) | 96.54 (90.01 - 98.49) | 96.64 (90.23 - 98.53) | 76.81 (61.92 - 84.92) | 97.18 (91.26 - 98.81) |
| MSM non-IDU | 81.92 (69.12 - 88.14) | 92.49 (83.53 - 95.86) | 93.92 (85.88 - 96.79) | 93.95 (85.96 - 96.80) | 69.65 (54.94 - 78.15) | 94.96 (87.65 - 97.45) |
| Ever MSM-IDU | 85.32 (72.54 - 91.27) | 94.31 (86.02 - 97.21) | 95.42 (87.95 - 97.87) | 95.86 (89.01 - 98.08) | 74.35 (58.81 - 82.69) | 96.23 (89.44 - 98.31) |

*Units for incidence and prevalence are /100 person-years and %, respectively.

Abbreviations: CrI=credible interval; HCNSP: high-coverage needle and syringe program; SOC=standard of care

## **Supplementary Table 9:** Results**—**Year when HCV incidence is estimated to decrease by 80% compared to 2015 levels

|  | **Status quo.**  No recovery in COVID-19 related disruptions | **Scenario 1:**  **Slow** recovery**.**  Recovery in COVID-19 related disruptions by 2025 | **Scenario 2:**  **Rapid** recovery**.**  Recovery in COVID-19 related disruptions by 2022 | **Scenario 3: Rapid plus.**  Scenario 2 + increase HCNSP to 100% | **Counterfactual:** Remove HCV testing and treatment over 2023-2030. | **Counterfactual:**  No COVID-19 |
| --- | --- | --- | --- | --- | --- | --- |
|  | **Median**  **(95% CrI)** | **Median**  **(95% CrI)** | **Median**  **(95% CrI)** | **Median**  **(95% CrI)** | **Median**  **(95% CrI)** | **Median**  **(95% CrI)** |
| MSM non-IDU | 2026  (2022 – 2035) | 2024  (2022 – 2028) | 2023  (2022-2027) | 2023  (2022-2027) | 2041*  (2021 – 2086) | 2022  (2021-2026) |
| Ever MSM-IDU | 2026  (2022 – 2037) | 2024  (2022 – 2028) | 2023  (2022-2027) | 2023  (2022-2026) | 2042*  (2022 – 2083) | 2023  (2021-2026) |

***** Most projections (840/1000 and 825/1000 for ever MSM-IDU and MSM non-IDU, respectively) do not decrease by 80% in 2030. The estimates in the figure are based on a subset of model projections that decreased by 80% if the model was run until 2090 (714/1000 and 534/1000 for ever MSM-IDU and MSM non-IDU, respectively).

Abbreviation: CrI= credible interval.

## **Supplementary Table 10:** Results**—**Estimated contribution of HCV testing and treatment to the decline in HCV incidence among MSM

|  | *Full* contribution of HCV testing and treatment over 2015-2022 | Contribution of *scaled-up* HCV testing and treatment over 2015-2022 | *Full* contribution of HCV testing and treatment over 2015-2030 | Contribution of *scaled-up* HCV testing and treatment over 2015-2030 |
| --- | --- | --- | --- | --- |
| Ever MSM-IDU | 86%  (80.5% - 94.9%) | 65.4%  (58.9% - 72.7%) | 75.8%  (66.7% - 89.5%) | 54.1%  (46.9% - 64.6%) |
| MSM non-IDU | 92.2%  (87.1% - 100%) | 69.6%  (63.2% - 75.4%) | 84.5%  (75.7% - 98.8%) | 59.3%  (51.9% - 68.9%) |

## **Supplementary Table 11:** Results – Modelled rates of HCV diagnosis and HCV treatment

|  | **Population** | **2010** | **2015** | **2030** |
| --- | --- | --- | --- | --- |
| **Rate of HCV diagnosis among chronically infected MSM ( /100py)** | | | | |
|  | All MSM | 18.5 (15.6 – 21.6) | 27.9 (23.1 – 31.9) | 14.8 (11.7 – 17.8) |
|  | Recent MSM-IDU | 26.2 (20.0 – 31.8) | 39.3 (30.0 – 47.7) | 18.3 (14.0 – 22.2) |
|  | Prior MSM-IDU | 26.2 (20.0 – 31.8) | 39.3 (30.0 – 47.7) | 18.3 (14.0 – 22.2) |
|  | Ever MSM-IDU | 26.2 (20.0 – 31.8) | 39.3 (30.0 -47.7) | 18.3 (14.0 – 22.2) |
|  | MSM non-IDU | 14.3 (12.0 – 15.9) | 22.8 (19.1 – 25.7) | 12.6 (10.1 – 14.9) |
| **Rate of HCV treatment among chronically infected and diagnosed MSM ( /100py)** | | | | |
|  | All MSM | 3.1 (2.8 – 3.5) | 29.4 (19.6 – 38.5) | 12.0 (8.0 – 15.8) |
|  | Recent MSM-IDU | 3.1 (2.8 – 3.5) | 29.4 (19.6 – 38.5) | 12.0 (8.0 – 15.8) |
|  | Prior MSM-IDU | 3.1 (2.8 – 3.5) | 29.4 (19.6 – 38.5) | 12.0 (8.0 – 15.8) |
|  | Ever MSM-IDU | 3.1 (2.8 – 3.5) | 29.4 (19.6 – 38.5) | 12.0 (8.0 – 15.8) |
|  | MSM non-IDU | 3.1 (2.8 – 3.5) | 29.4 (19.6 – 38.5) | 12.0 (8.0 – 15.8) |

## **Supplementary Table 12:** Results—Number and proportion of incident HCV cases averted over 2023-2030 compared to a scenario in which the standard of care is removed over 2023-2030 (scenario 4)

|  | **Status quo.**  No recovery in COVID-19 related disruptions | **Scenario 1:**  **Slow** recovery**.**  Recovery in COVID-19 related disruptions by 2025 | **Scenario 2:**  **Rapid** recovery**.**  Recovery in COVID-19 related disruptions by 2022 | **Scenario 3: Rapid plus.**  Scenario 2 + increase HCNSP to 100% | **Counterfactual:** Remove HCV testing and treatment over 2023-2030. | **Counterfactual:**  No COVID-19 |
| --- | --- | --- | --- | --- | --- | --- |
|  | **Median (95% CrI)** | **Median (95% CrI)** | **Median (95% CrI)** | **Median (95% CrI)** | **Median (95% CrI)** | **Median (95% CrI)** |
| **Cumulative number of incident HCV cases over 2023-2030** | | | | | | |
| MSM non-IDU | 130 (74 – 235) | 89 (48 – 176) | 74 (38 – 153) | 73 (38 – 151) | 168 (101 – 286) | 61 (31 – 133) |
| Ever MSM-IDU | 138 (72 – 274) | 97 (49 – 211) | 80 (40 – 184) | 66 (33 – 148) | 173 (94 – 322) | 68 (32 – 162) |
| **Cumulative number of incident HCV cases averted over 2023-2030** | | | | | | |
| MSM non-IDU | 38 (26 – 53) | 79 (52 – 113) | 94 (61 -136) | 95 (62 -138) | REF | 106 (69 – 155) |
| Ever MSM-IDU | 34 (22 – 52) | 74 (46 – 119) | 90 (55 – 146) | 106 (61 – 180) | REF | 103 (62 – 169) |
| **Proportion of incident HCV cases averted over 2023-2030 (%)** | | | | | | |
| MSM non-IDU | 23 (17 – 27) | 47 (37 – 53) | 56 (45 – 63) | 56 (45 – 63) | REF | 63 (51 – 70) |
| Ever MSM-IDU | 20 (14 – 24) | 44 (33 – 50) | 53 (41 – 60) | 61 (52 – 67) | REF | 60 (48 – 68) |

Abbreviation: CrI= credible interval

## **Supplementary Table 13:** Results**—**Posterior ranges for parameters included in the model

| **Parameter** | **Min** | **Median** | **Max** |
| --- | --- | --- | --- |
| MSM population size in 1985 | 123250.0000 | 132560.0000 | 139980.0000 |
| HIV seed in 1985 among all MSM | 0.3611 | 0.4123 | 0.4643 |
| HCV seed among ever MSM-IDU in 1985 | 0.1189 | 0.1898 | 0.2517 |
| HCV seed among MSM non-IDU in 1985 | 0.0092 | 0.0207 | 0.0286 |
| Proportion of HIV-infected MSM co-infected with HCV | 0.3738 | 0.6665 | 0.9399 |
| Relative risk of HCV transmission through injection drug use practices vs sexual practices | 2.0004 | 2.7796 | 3.8338 |
| Sexually-related HCV transmission rate (per person per year) | 0.0203 | 0.0283 | 0.0383 |
| Relative risk of HCV infectivity among MSM who are HIV-infected vs MSM who are not HIV-infected | 1.5057 | 2.0864 | 2.7786 |
| Proportion of individuals who spontaneously clear HCV infection among HIV negative MSM | 0.2202 | 0.2408 | 0.2597 |
| Proportion of individuals who spontaneously clear HCV infection among HIV positive MSM | 0.1202 | 0.1686 | 0.1900 |
| Proportion of MSM non-IDU, who were HIV-negative and not on PrEP and reported HCV testing in the past year | 0.1333 | 0.1729 | 0.1988 |
| Relative risk of HCV testing among MSM who belonged to any of the following groups, compared to MSM non-IDU who were HIV-negative and not on PrEP: ever MSM-IDU or HIV negative MSM on PrEP or HIV positive MSM | 1.8731 | 2.3670 | 2.9006 |
| Proportion of HIV-positive MSM who reported ever treatment over 2008-2014 | 0.1344 | 0.1539 | 0.1789 |
| Relative risk reflecting the increase in rate of HCV treatment in 2015 due to DAA scale-up relative to previous years | 3.9837 | 7.0012 | 9.8251 |
| Proportion of HCV treatments that result in SVR during the interferon era (2004-2014) among HIV-negative MSM | 0.6069 | 0.6372 | 0.6700 |
| Proportion of HCV treatments that result in SVR during the PEG-IFN era (2004-2014) among HIV-positive MSM | 0.3721 | 0.3810 | 0.3884 |
| Proportion of HCV treatments that result in SVR during the DAA era (2015-onward) irrespective of HIV status | 0.9384 | 0.9748 | 0.9996 |
| Year HCV testing started | 1999.0000 | 1999.2000 | 1999.5000 |
| Year interferon-based HCV treatment started | 2002.0000 | 2002.6000 | 2003.3000 |
| Rate of exit from the HCV treatment compartment during the DAA era (2015-onward; per person per year) | 4.3008 | 5.1308 | 6.1861 |
| HCV-related mortality among MSM who are not infected with HIV (per person per year) | 0.0021 | 0.0049 | 0.0076 |
| Relative risk of HCV-related mortality among MSM who are co-infected with HIV | 1.6935 | 1.9709 | 2.2647 |
| Injection drug use-related HIV transmission rate (per person per year) | 0.0255 | 0.0375 | 0.0592 |
| Sexually-related HIV transmission rate (per person per year) | 0.0982 | 0.1108 | 0.1234 |
| Proportion of diagnosed MSM on ART in 2017 | 0.8935 | 0.9347 | 0.9771 |
| Proportion of diagnosed MSM on ART in 2008 | 0.7628 | 0.7880 | 0.8158 |
| Relative risk reflecting reduced HIV infectivity among MSM on ART vs those who are not on ART | 0.0000 | 0.0000 | 0.0000 |
| Relative risk reflecting the reduced susceptibility to injection drug use-related HIV acquisition among MSM on PrEP vs those not on PrEP | 0.3267 | 0.4518 | 0.6064 |
| Relative risk reflecting the reduced susceptibility to sexually-related HIV acquisition among MSM on PrEP vs those not on PrEP | 0.0805 | 0.1638 | 0.2545 |
| Rate at which HIV-negative susceptible individuals start using PrEP over 2010-2011 (per person per year) | 0.0397 | 0.0848 | 0.1051 |
| Rate at which HIV-negative susceptible individuals start using PrEP over 2011-2014 (per person per year) | 0.0146 | 0.1233 | 0.2258 |
| Rate at which HIV-negative susceptible individuals start using PrEP over 2014-2019 (per person per year) | 1.2786 | 1.5389 | 1.8407 |
| Rate at which HIV-negative susceptible individuals stop using PrEP (per person per year) | 1.3247 | 1.4833 | 1.6262 |
| Rate at which individuals are tested for HIV (per person per year) | 0.3381 | 0.4293 | 0.5083 |
| HIV-related mortality if MSM are not on ART (per person per year) | 0.0820 | 0.0835 | 0.0859 |
| Relative risk of HIV-related mortality among MSM who are on ART vs MSM who are not on ART | 0.2563 | 0.3120 | 0.3475 |
| The rate at which MSM initiate injection drug use (per person per year) | 0.0057 | 0.0070 | 0.0084 |
| Rate at which MSM stop injection drug use (per person per year) | 0.0301 | 0.0510 | 0.0755 |
| Injection drug use-related mortality (per person per year) | 0.0085 | 0.0248 | 0.0364 |

## **Supplementary Table 14:** Results**—**Uncertainty analyses

| **Parameter** | **% of the variance explained** |
| --- | --- |
| Relative risk reflecting the increase in rate of HCV treatment in 2015 due to DAA scale-up relative to previous years | 74.10 |
| Proportion of HIV-positive MSM who reported ever treatment over 2008-2014 | 9.57 |
| Proportion of MSM non-IDU, who were HIV-negative and not on PrEP and reported HCV testing in the past year | 4.88 |
| Relative risk of HCV testing among MSM who belonged to any of the following groups, compared to MSM non-IDU who were HIV-negative and not on PrEP: ever MSM-IDU or HIV negative MSM on PrEP or HIV positive MSM | 2.64 |
| Injection drug use-related HIV transmission rate (per person per year) | 2.60 |
| Relative risk of HCV transmission through injection drug use practices vs sexual practices | 2.52 |
| Rate at which MSM stop injection drug use (per person per year) | 1.03 |
| Injection drug use-related mortality (per person per year) | 0.48 |
| Proportion of HCV treatments that result in SVR during the DAA era (2015-onward) irrespective of HIV status | 0.42 |
| Proportion of individuals who spontaneously clear HCV infection among HIV positive MSM | 0.41 |
| Proportion of individuals who spontaneously clear HCV infection among HIV negative MSM | 0.32 |
| HCV seed among ever MSM-IDU in 1985 | 0.15 |
| Sexually-related HCV transmission rate (per person per year) | 0.14 |
| HCV-related mortality among MSM who are not infected with HIV (per person per year) | 0.13 |
| Relative risk reflecting the reduced susceptibility to sexually-related HIV acquisition among MSM on PrEP vs those not on PrEP | 0.08 |
| Proportion of diagnosed MSM on ART in 2008 | 0.08 |
| Proportion of diagnosed MSM on ART in 2017 | 0.06 |
| Year HCV testing started | 0.05 |
| Sexually-related HIV transmission rate (per person per year) | 0.05 |
| MSM population size in 1985 | 0.05 |
| The rate at which MSM initiate injection drug use (per person per year) | 0.03 |
| Year interferon-based HCV treatment started | 0.03 |
| HIV seed in 1985 among all MSM | 0.03 |
| HIV-related mortality if MSM are not on ART (per person per year) | 0.02 |
| Rate at which HIV-negative susceptible individuals start using PrEP over 2010-2011 (per person per year) | 0.02 |
| Relative risk of HCV-related mortality among MSM who are co-infected with HIV | 0.02 |
| HCV seed among MSM non-IDU in 1985 | 0.02 |
| Proportion of HCV treatments that result in SVR during the interferon era (2004-2014) among HIV-negative MSM | 0.02 |
| Proportion of HIV-infected MSM co-infected with HCV | 0.01 |
| Relative risk reflecting the reduced susceptibility to injection drug use-related HIV acquisition among MSM on PrEP vs those not on PrEP | 0.01 |
| Rate at which HIV-negative susceptible individuals start using PrEP over 2014-2019 (per person per year) | 0.01 |
| Proportion of HCV treatments that result in SVR during the PEG-IFN era (2004-2014) among HIV-positive MSM | 0.01 |
| Rate of exit from the HCV treatment compartment during the DAA era (2015-onward; per person per year) | 0.01 |
| Rate at which HIV-negative susceptible individuals stop using PrEP (per person per year) | 0.00 |
| Relative risk of HIV-related mortality among MSM who are on ART vs MSM who are not on ART | 0.00 |
| Relative risk of HCV infectivity among MSM who are HIV-infected vs MSM who are not HIV-infected | 0.00 |
| Rate at which individuals are tested for HIV (per person per year) | 0.00 |
| Rate at which HIV-negative susceptible individuals start using PrEP over 2011-2014 (per person per year) | 0.00 |
| Relative risk reflecting reduced HIV infectivity among MSM on ART vs those who are not on ART | 0.00 |

# **SUPPLEMENTARY FIGURES**

## **Supplementary Figure 1:** Model fit to calibration data for (A) MSM population size and (B) HIV-negative ever MSM-IDU

**
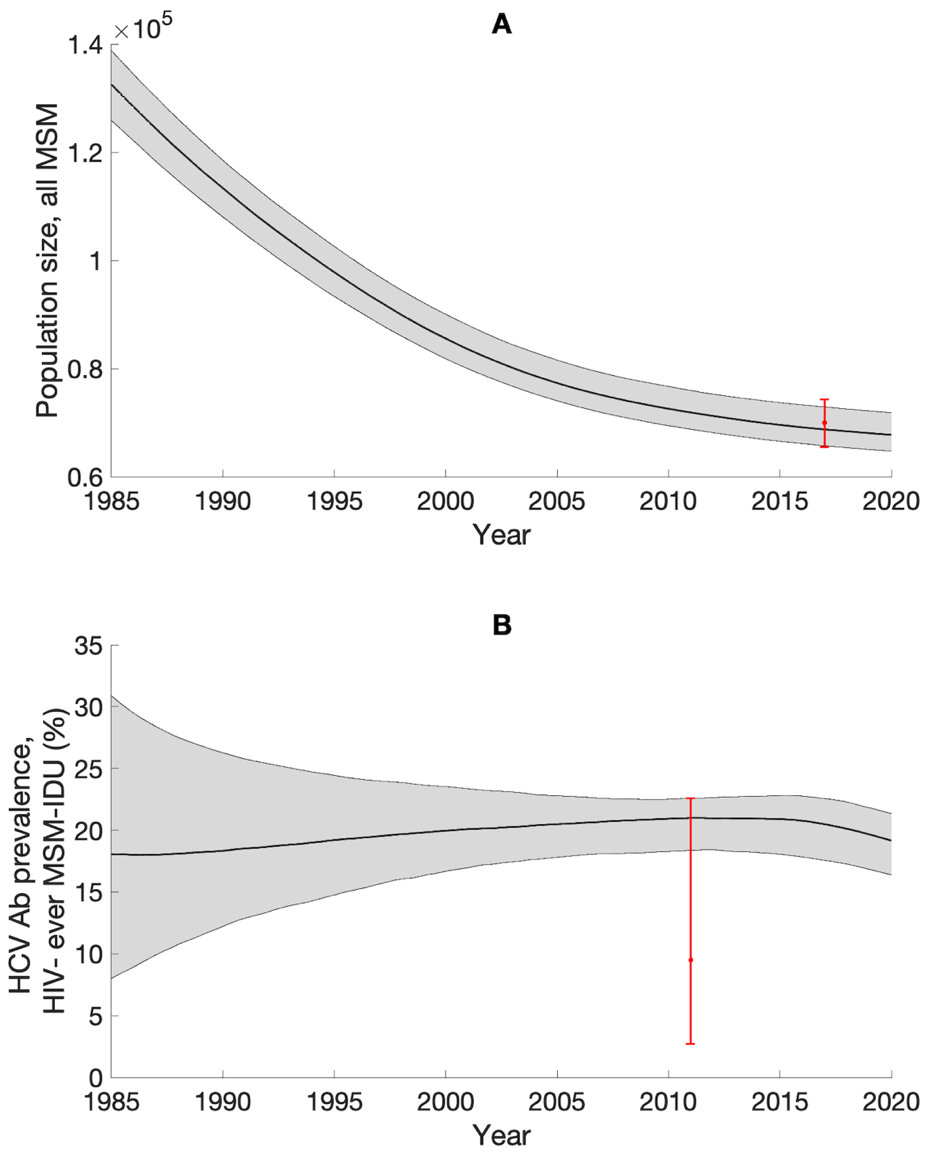
**

## **Supplementary Figure 2:** Model fit to calibration data on proportion of HCV-diagnosed MSM who were ever treated


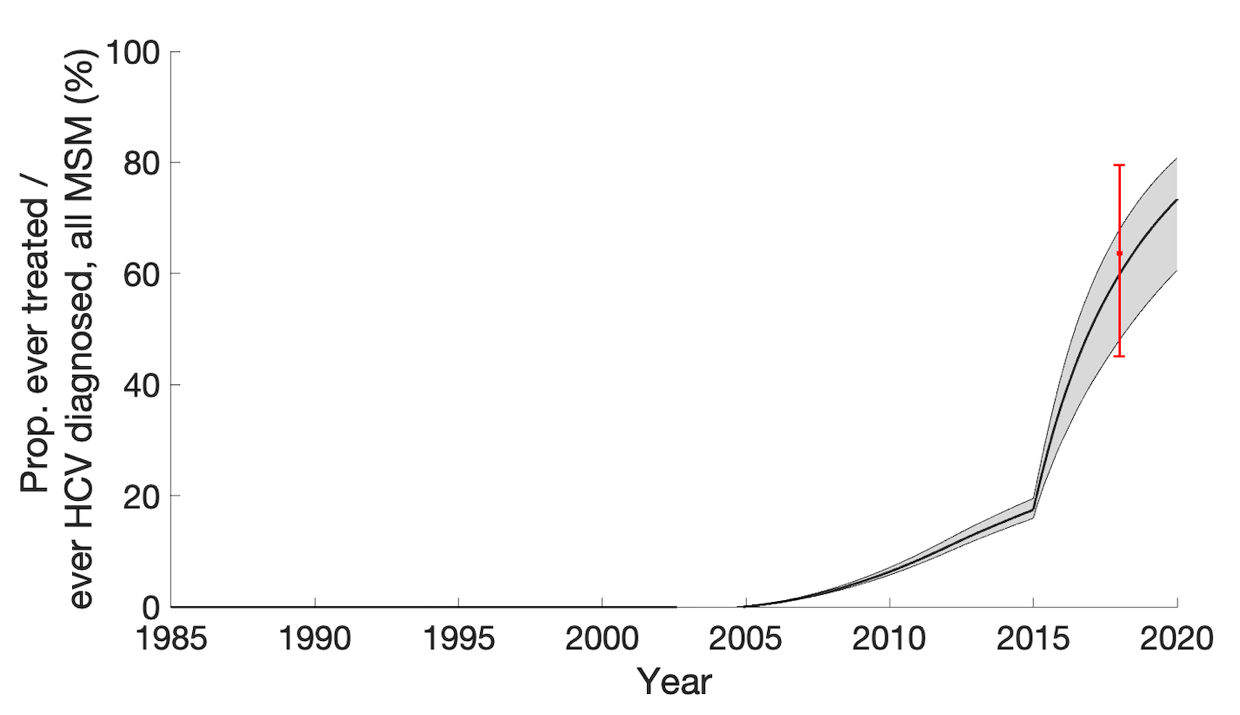


## **Supplementary Figure 3:** Model fit to calibration data on proportion of HIV-positive MSM who are diagnosed


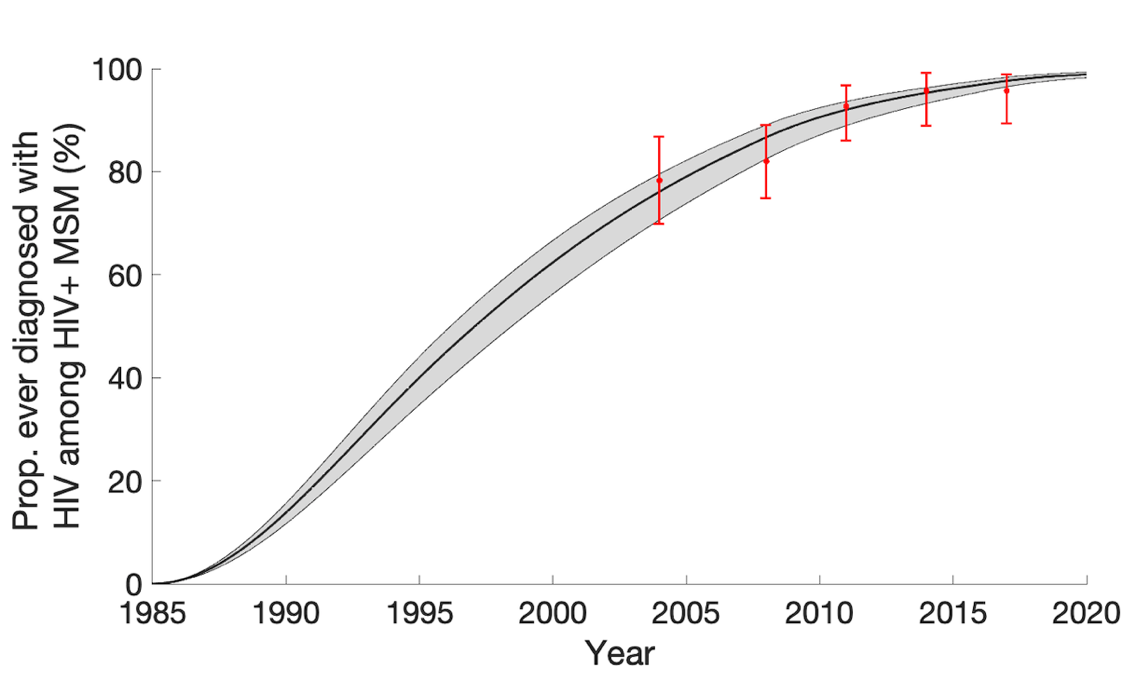


## **Supplementary Figure 4:** Model fit to validation data on proportion of MSM who injected in the previous year (recent MSM-IDU)


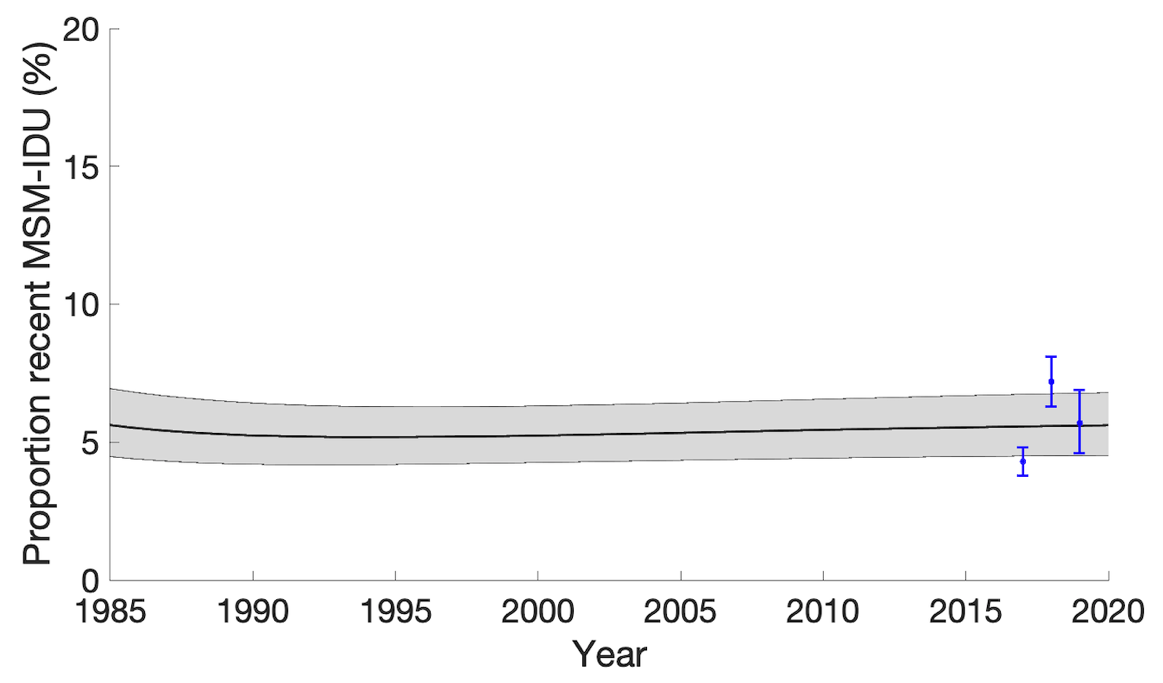


## **Supplementary Figure 5:** Model fit to validation data on proportion of MSM ever chronically infected with HCV who were ever diagnosed


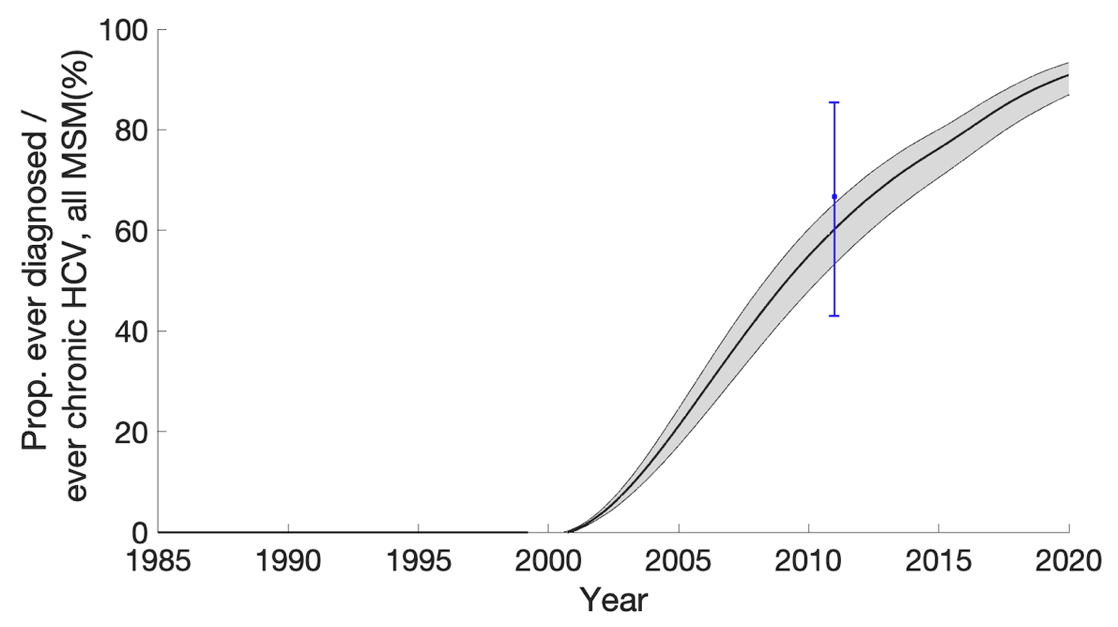


## **Supplementary Figure 6:** Model fit to validation data on HCV Ab prevalence among (A) all MSM, (B) HIV+ ever MSM-IDU, (C) HIV+ MSM non-IDU and (D) HIV- MSM non-IDU

**
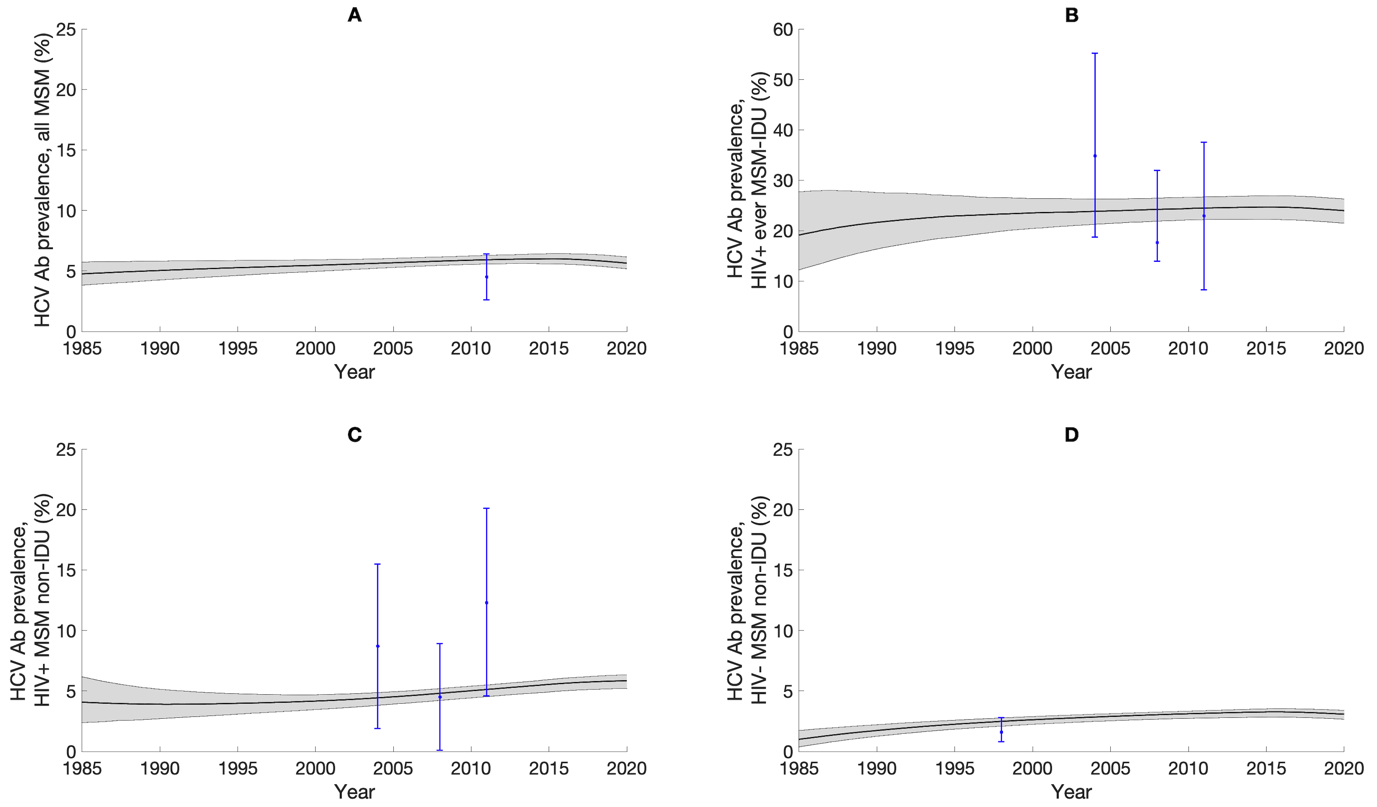
**

## **Supplementary Figure 7:** Model fit to validation data on HIV prevalence, all MSM


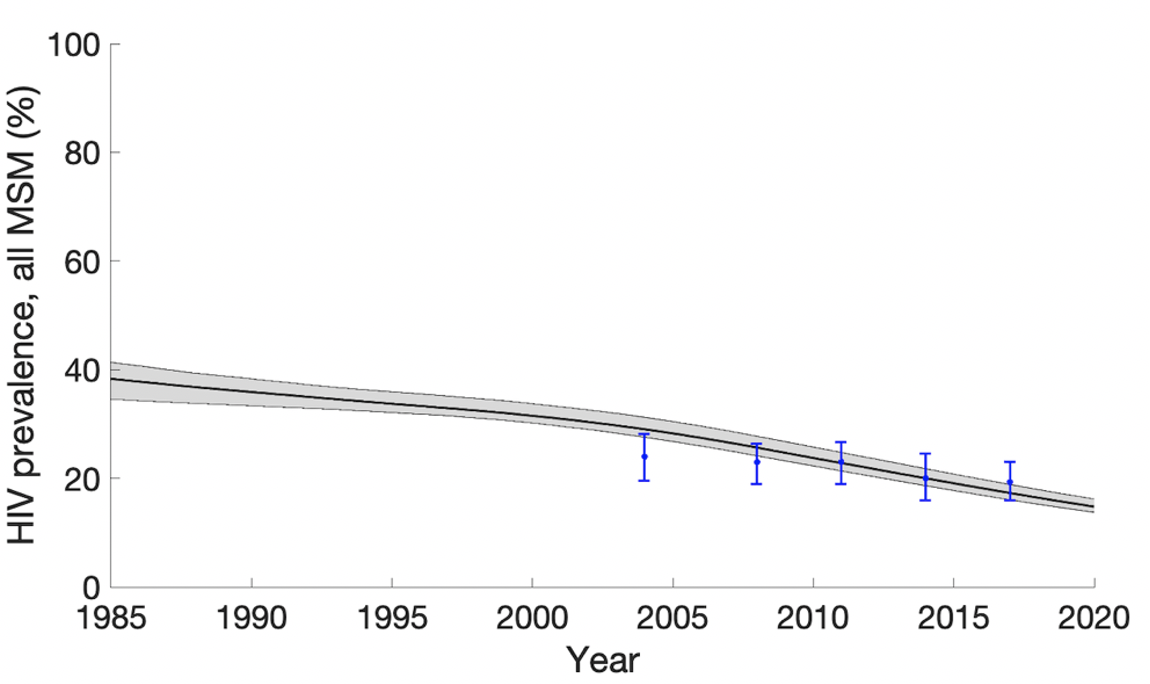


## **Supplementary Figure 8:** Projected year when HCV incidence decreases by 80% compared to 2015 levels among ever MSM-IDU and MSM non-IDU, for different scenarios

**
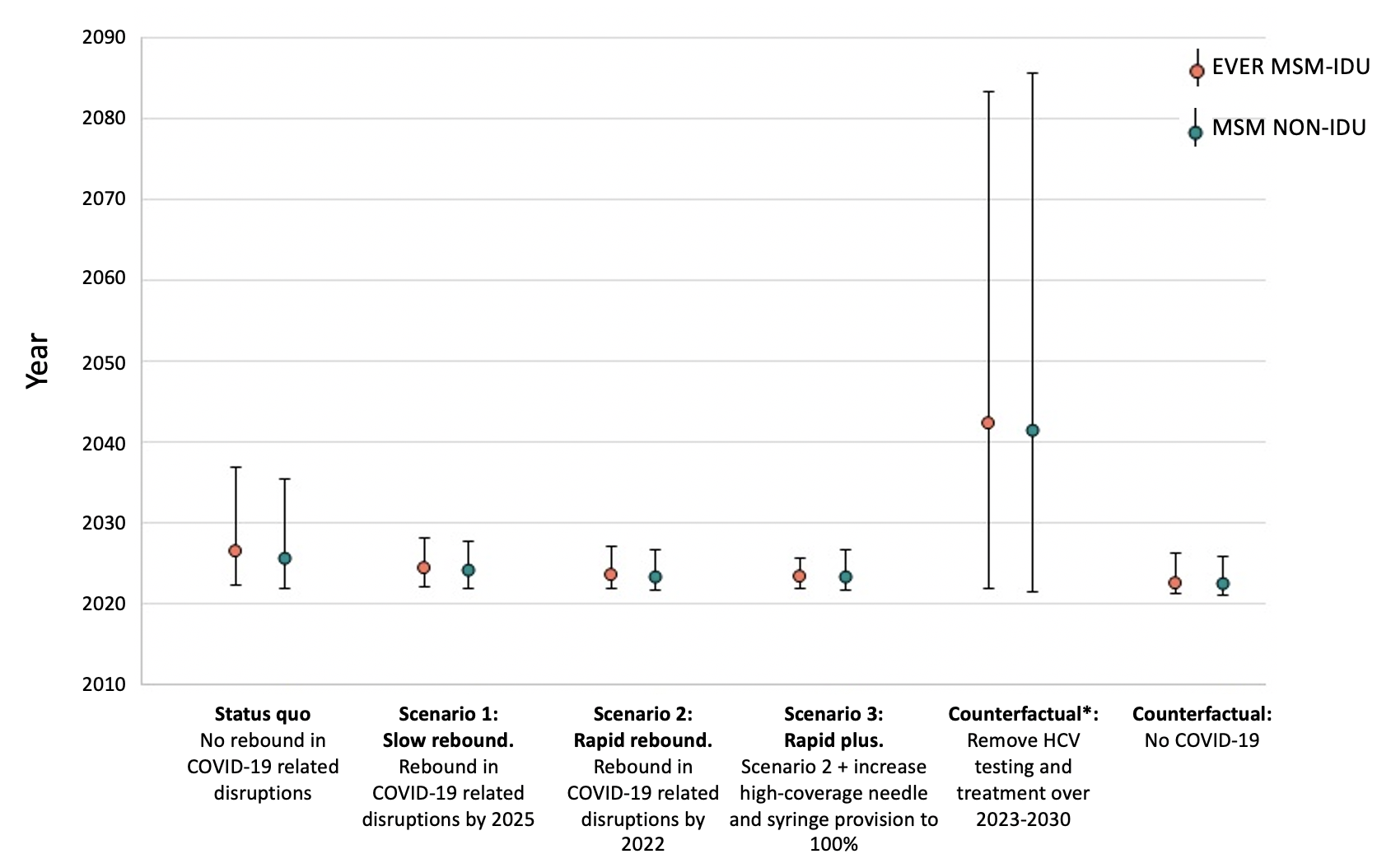
**

**Notes:** Dots represent the median value and whiskers represent 2.5th and 97.5th percentiles. *In this counterfactual, most projections (840/1000 and 825/1000 for ever MSM-IDU and MSM non-IDU, respectively) do not decrease by 80% in 2030. The estimates shown in the figure are based on a subset of model projections (714/1000 and 534/1000 for ever MSM-IDU and MSM non-IDU, respectively) that decreased by 80% if the model was run until 2090.

## **Supplementary Figure 9:** Proportion of incident cases of HCV infection averted over 2023-2030 among ever MSM-IDU and MSM non-IDU for different scenarios, compared to a scenario in which there is no HCV testing and treatment over 2023-2030


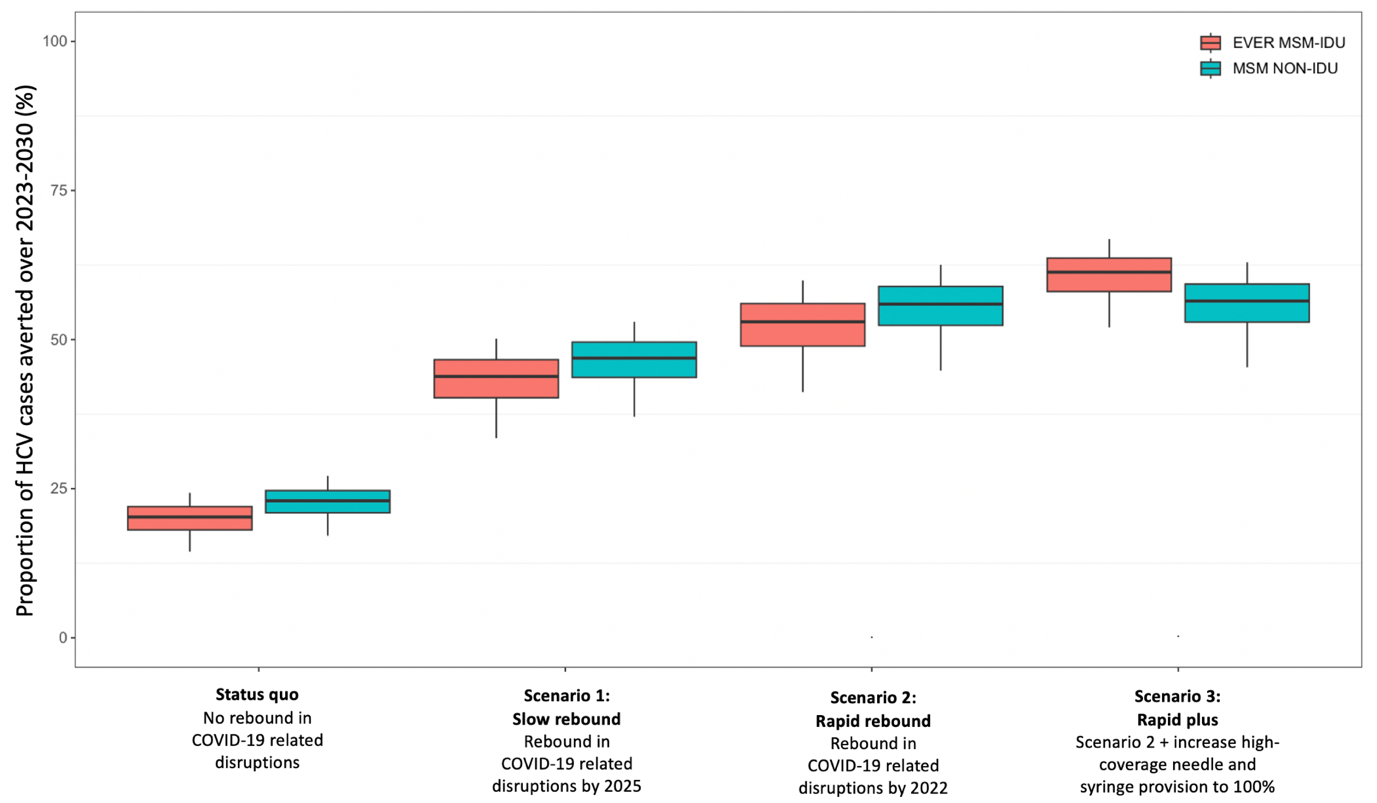


**Note:** Boxes represent the median and 25th - 75th percentile range and whiskers represent 2.5th and 97.5th percentiles.

REFERENCES

1. Zhou K, Terrault NA. Gaps in Viral Hepatitis Awareness in the United States in a Population-based Study. Clinical Gastroenterology and Hepatology **2020**; 18(1): 188-95.e4.

2. Centers for Disease Control and Prevention. Testing Recommendations for Hepatitis C Virus Infection [web page]. 2020. Available at: <https://www.cdc.gov/hepatitis/hcv/guidelinesc.htm>. Accessed October 25, 2021.

3. Raymond HF, Hughes A, O'Keefe K, Stall RD, McFarland W. Hepatitis C prevalence among HIV-positive MSM in San Francisco: 2004 and 2008. Sex Transm Dis **2011**; 38(3): 219-20.

4. Raymond HF, Chu P, Nieves-Rivera I, Louie B, McFarland W, Pandori M. Hepatitis C infection among men who have sex with men, San Francisco, 2011. Sex Transm Dis **2012**; 39(12): 985-6.

5. Cachay ER, Hill L, Wyles D, et al. The hepatitis C cascade of care among HIV infected patients: a call to address ongoing barriers to care. PLoS One **2014**; 9(7): e102883.

6. Johnson TL, Toliver JC, Mao L, Oramasionwu CU. Differences in outpatient care and treatment utilization for patients with HIV/HCV coinfection, HIV, and HCV monoinfection, a cross-sectional study. BMC Infect Dis **2014**; 14: 217.

7. Barnett PG, Joyce VR, Lo J, et al. Effect of Interferon-Free Regimens on Disparities in Hepatitis C Treatment of US Veterans. Value Health **2018**; 21(8): 921-30.

8. Cheung R, Mannalithara A, Singh G. Utilization and antiviral therapy in patients with chronic hepatitis C: analysis of ambulatory care visits in the US. Dig Dis Sci **2010**; 55(6): 1744-51.

9. Centers for Disease Control and Prevention. New estimates reveal declines in hepatitis C treatment in the US between 2015 and 2020. Atlanta, GA, 2021. Available at: <https://www.cdc.gov/nchhstp/newsroom/2021/2014-2020-hepatitis-c-treatment-estimates.html>. Accessed Jan 15, 2021.

10. Centers for Disease Control and Prevention. US Public Health Service. Preexposure prophylaxis for the prevention of HIV infection in the United States - 2021 update. A clinical practice guideline.

11. Raymond HF, Chen YH, Ick T, et al. A new trend in the HIV epidemic among men who have sex with men, San Francisco, 2004-2011. J Acquir Immune Defic Syndr **2013**; 62(5): 584-9.

12. HIV.org. A Timeline of HIV and AIDS. 2020. Available at: <https://www.hiv.gov/hiv-basics/overview/history/hiv-and-aids-timeline>. Accessed November 2021.

13. Chen YH, Guigayoma J, McFarland W, Snowden JM, Raymond HF. Increases in Pre-exposure Prophylaxis Use and Decreases in Condom Use: Behavioral Patterns Among HIV-Negative San Francisco Men Who have Sex with Men, 2004-2017. AIDS Behav **2019**; 23(7): 1841-5.

14. Centers for Disease Control and Prevention. National HIV Behavioral Surveillance (NHBS). Atlanta, GA, 2021. Available at: <https://www.cdc.gov/hiv/statistics/systems/nhbs/reports.html>. Accessed October 2021.

15. Facente SN, Patel S, Hecht J, et al. Hepatitis C care cascades for three populations at high risk: low-income trans women, young people who inject drugs, and men who have sex with men and inject drugs. Clinical Infectious Diseases **2021**.

16. Bhatta DN, Hecht J, Facente SN. Psychosocial Determinants of HIV Stigma among Men Who Have Sex with Men in San Francisco, California. Int J Environ Res Public Health **2021**; 18(15).

17. Clipman SJ, Duggal P, Srikrishnan AK, et al. Prevalence and Phylogenetic Characterization of Hepatitis C Virus Among Indian Men Who Have Sex With Men: Limited Evidence for Sexual Transmission. J Infect Dis **2020**; 221(11): 1875-83.

18. Hoornenborg E, Coyer L, Boyd A, et al. High incidence of HCV in HIV-negative men who have sex with men using pre-exposure prophylaxis. Journal of Hepatology **2020**; 72(5): 855-64.

19. Mata-Marín JA, de Pablos-Leal AA, Mauss S, et al. Risk factors for HCV transmission in HIV-positive men who have sex with men in México. PLOS ONE **2022**; 17(7): e0269977.

20. Vanhommerig JW, Lambers FA, Schinkel J, et al. Risk Factors for Sexual Transmission of Hepatitis C Virus Among Human Immunodeficiency Virus-Infected Men Who Have Sex With Men: A Case-Control Study. Open Forum Infect Dis **2015**; 2(3): ofv115.

21. Witt MD, Seaberg EC, Darilay A, et al. Incident hepatitis C virus infection in men who have sex with men: a prospective cohort analysis, 1984-2011. Clin Infect Dis **2013**; 57(1): 77-84.

22. Facente SN, Grebe E, Burk K, et al. Estimated hepatitis C prevalence and key population sizes in San Francisco: A foundation for elimination. PLoS One **2018**; 13(4): e0195575.

23. Mathers BM, Degenhardt L. Examining non-AIDS mortality among people who inject drugs. AIDS **2014**; 28 Suppl 4: S435-44.

24. Mathers BM, Degenhardt L, Bucello C, Lemon J, Wiessing L, Hickman M. Mortality among people who inject drugs: a systematic review and meta-analysis. Bull World Health Organ **2013**; 91(2): 102-23.

25. Artenie A, Facente SN, Patel S, et al. A cross-sectional study comparing men who have sex with men and inject drugs and people who inject drugs who are men and have sex with men in San Francisco: Implications for HIV and hepatitis C virus prevention. Health Sci Rep **2022**; 5(4): e704.

26. National Vital Statistics Reports. Deaths: Final Data for 2019. National Center for Health Statistics, 2021. Available at: <https://www.cdc.gov/nchs/data/nvsr/nvsr70/nvsr70-08-508.pdf>. Accessed Dec 4th, 2021.

27. Time from HIV-1 seroconversion to AIDS and death before widespread use of highly-active antiretroviral therapy: a collaborative re-analysis. Collaborative Group on AIDS Incubation and HIV Survival including the CASCADE EU Concerted Action. Concerted Action on SeroConversion to AIDS and Death in Europe. Lancet **2000**; 355(9210): 1131-7.

28. May MT, Gompels M, Delpech V, et al. Impact on life expectancy of HIV-1 positive individuals of CD4+ cell count and viral load response to antiretroviral therapy. AIDS **2014**; 28(8): 1193-202.

29. El-Kamary SS, Jhaveri R, Shardell MD. All-cause, liver-related, and non-liver-related mortality among HCV-infected individuals in the general US population. Clin Infect Dis **2011**; 53(2): 150-7.

30. Thein HH, Yi Q, Dore GJ, Krahn MD. Natural history of hepatitis C virus infection in HIV-infected individuals and the impact of HIV in the era of highly active antiretroviral therapy: a meta-analysis. AIDS **2008**; 22(15): 1979-91.

31. Osmond DH, Charlebois E, Sheppard HW, et al. Comparison of risk factors for hepatitis C and hepatitis B virus infection in homosexual men. J Infect Dis **1993**; 167(1): 66-71.

32. Macgregor L, Desai M, Martin NK, et al. Scaling up screening and treatment for elimination of hepatitis C among men who have sex with men in the era of HIV pre-exposure prophylaxis. EClinicalMedicine **2020**; 19.

33. MacGregor L, Martin NK, Mukandavire C, et al. Behavioural, not biological, factors drive the HCV epidemic among HIV-positive MSM: HCV and HIV modelling analysis including HCV treatment-as-prevention impact. Int J Epidemiol **2017**; 46(5): 1582-92.

34. Vickerman P, Platt L, Hawkes S. Modelling the transmission of HIV and HCV among injecting drug users in Rawalpindi, a low HCV prevalence setting in Pakistan. Sex Transm Infect **2009**; 85 Suppl 2: ii23-30.

35. Benova L, Mohamoud YA, Calvert C, Abu-Raddad LJ. Vertical transmission of hepatitis C virus: systematic review and meta-analysis. Clin Infect Dis **2014**; 59(6): 765-73.

36. De Carli G, Puro V, Ippolito G, Studio Italiano Rischio Occupazionale da HIVG. Risk of hepatitis C virus transmission following percutaneous exposure in healthcare workers. Infection **2003**; 31 Suppl 2: 22-7.

37. Yazdanpanah Y, De Carli G, Migueres B, et al. Risk factors for hepatitis C virus transmission to health care workers after occupational exposure: a European case-control study. Clin Infect Dis **2005**; 41(10): 1423-30.

38. Thomas DL, Astemborski J, Vlahov D, et al. Determinants of the quantity of hepatitis C virus RNA. J Infect Dis **2000**; 181(3): 844-51.

39. Thomas DL, Rich JD, Schuman P, et al. Multicenter evaluation of hepatitis C RNA levels among female injection drug users. J Infect Dis **2001**; 183(6): 973-6.

40. Bonacini M, Lin HJ, Hollinger FB. Effect of coexisting HIV-1 infection on the diagnosis and evaluation of hepatitis C virus. J Acquir Immune Defic Syndr **2001**; 26(4): 340-4.

41. Daar ES, Lynn H, Donfield S, et al. Relation between HIV-1 and hepatitis C viral load in patients with hemophilia. J Acquir Immune Defic Syndr **2001**; 26(5): 466-72.

42. Fishbein DA, Lo Y, Netski D, Thomas DL, Klein RS. Predictors of hepatitis C virus RNA levels in a prospective cohort study of drug users. J Acquir Immune Defic Syndr **2006**; 41(4): 471-6.

43. Micallef JM, Kaldor JM, Dore GJ. Spontaneous viral clearance following acute hepatitis C infection: a systematic review of longitudinal studies. J Viral Hepat **2006**; 13(1): 34-41.

44. Smith DJ, Jordan AE, Frank M, Hagan H. Spontaneous viral clearance of hepatitis C virus (HCV) infection among people who inject drugs (PWID) and HIV-positive men who have sex with men (HIV+ MSM): a systematic review and meta-analysis. BMC Infect Dis **2016**; 16: 471.

45. Panel A-IHG. Hepatitis C Guidance 2018 Update: AASLD-IDSA Recommendations for Testing, Managing, and Treating Hepatitis C Virus Infection. Clinical Infectious Diseases **2018**; 67(10): 1477-92.

46. Davies A, Singh KP, Shubber Z, et al. Treatment outcomes of treatment-naïve Hepatitis C patients co-infected with HIV: a systematic review and meta-analysis of observational cohorts. PLoS One **2013**; 8(2): e55373.

47. American Association for the Study of Liver Diseases IDSoA. HCV Guidance: Recommendations for Testing, Managing, and Treating Hepatitis C. Available at: <https://www.hcvguidelines.org>. Accessed July 7, 2020.

48. Borroni G, Andreoletti M, Casiraghi MA, et al. Effectiveness of pegylated interferon/ribavirin combination in 'real world' patients with chronic hepatitis C virus infection. Aliment Pharmacol Ther **2008**; 27(9): 790-7.

49. Scotto R, Buonomo AR, Moriello NS, et al. Real-World Efficacy and Safety of Pangenotypic Direct-Acting Antivirals Against Hepatitis C Virus Infection. Rev Recent Clin Trials **2019**; 14(3): 173-82.

50. Winkelstein W, Jr., Samuel M, Padian NS, et al. The San Francisco Men's Health Study: III. Reduction in human immunodeficiency virus transmission among homosexual/bisexual men, 1982-86. Am J Public Health **1987**; 77(6): 685-9.

51. Rodger AJ, Cambiano V, Bruun T, et al. Risk of HIV transmission through condomless sex in serodifferent gay couples with the HIV-positive partner taking suppressive antiretroviral therapy (PARTNER): final results of a multicentre, prospective, observational study. Lancet **2019**; 393(10189): 2428-38.

52. Cohen MS, Chen YQ, McCauley M, et al. Antiretroviral Therapy for the Prevention of HIV-1 Transmission. N Engl J Med **2016**; 375(9): 830-9.

53. Choopanya K, Martin M, Suntharasamai P, et al. Antiretroviral prophylaxis for HIV infection in injecting drug users in Bangkok, Thailand (the Bangkok Tenofovir Study): a randomised, double-blind, placebo-controlled phase 3 trial. Lancet **2013**; 381(9883): 2083-90.

54. Chou R, Evans C, Hoverman A, et al. Preexposure Prophylaxis for the Prevention of HIV Infection: Evidence Report and Systematic Review for the US Preventive Services Task Force. JAMA **2019**; 321(22): 2214-30.

55. Centers for Disease Control and Prevention. CDC Statement on FDA Approval of Drug for HIV Prevention. 2012. Available at: <https://www.cdc.gov/nchhstp/newsroom/2012/fda-approvesdrugstatement.html>. Accessed March 28, 2022.

56. Spinelli MA, Scott HM, Vittinghoff E, et al. Missed Visits Associated With Future Preexposure Prophylaxis (PrEP) Discontinuation Among PrEP Users in a Municipal Primary Care Health Network. Open Forum Infect Dis **2019**; 6(4): ofz101.

57. Raymond HF, McFarland W, Wesson P. Estimated Population Size of Men Who Have Sex with Men, San Francisco, 2017. AIDS Behav **2019**; 23(6): 1576-9.

58. Hammer GP, Kellogg TA, McFarland WC, et al. Low incidence and prevalence of hepatitis C virus infection among sexually active non-intravenous drug-using adults, San Francisco, 1997-2000. Sex Transm Dis **2003**; 30(12): 919-24.

59. Winkelstein W, Jr., Wiley JA, Padian NS, et al. The San Francisco Men's Health Study: continued decline in HIV seroconversion rates among homosexual/bisexual men. Am J Public Health **1988**; 78(11): 1472-4.

60. Katz MH, Schwarcz SK, Kellogg TA, et al. Impact of highly active antiretroviral treatment on HIV seroincidence among men who have sex with men: San Francisco. Am J Public Health **2002**; 92(3): 388-94.

61. Facente SN, Grinstein R, Broussard J, et al. Hepatitis C Elimination During a Global Pandemic: A Case Study of Resilience in Action. Public Health Rep **2022**: 333549221083741.

62. Hoenigl M, Abramovitz D, Flores Ortega RE, Martin NK, Reau N. Sustained impact of the COVID-2019 pandemic on HCV treatment initiations in the United States. Clin Infect Dis **2022**.

63. EndHepC SF. Ending the Epidemics. Collective strategies for addressing HIV, hepatitis C and sexually transmitted infections in San Francisco. 2020. Available at: <https://www.facenteconsulting.com/presentations/SF_ETE_Plan_FINAL.pdf>. Accessed Feb 23, 2022.

64. Huang YA, Zhu W, Wiener J, Kourtis AP, Hall HI, Hoover KW. Impact of COVID-19 on HIV Preexposure Prophylaxis Prescriptions in the United States - A Time Series Analysis. Clin Infect Dis **2022**.
